# Dissecting genetics of cutaneous miRNA in a mouse model of an autoimmune blistering disease

**DOI:** 10.1186/s12864-016-2455-2

**Published:** 2016-02-16

**Authors:** Yask Gupta, Steffen Möller, Mareike Witte, Meriem Belheouane, Tanya Sezin, Misa Hirose, Artem Vorobyev, Felix Niesar, Julia Bischof, Ralf J. Ludwig, Detlef Zillikens, Christian D. Sadik, Tobias Restle, Robert Häsler, John F. Baines, Saleh M. Ibrahim

**Affiliations:** Lübeck Institute of Experimental Dermatology, University of Lübeck, Lübeck, Germany; Department of Dermatology, University of Lübeck, Lübeck, Germany; Max Planck Institute for Evolutionary Biology, Plön, Germany and Institute for Experimental Medicine, University of Kiel, Kiel, Germany; Institute of Molecular Medicine, University of Lübeck, Lübeck, Germany; IKMB, Molecular Cell Biology, Kiel, Germany

**Keywords:** MicroRNA, Expression QTL, Epistasis, Autoimmune skin blistering disease, Co-expression analysis

## Abstract

**Background:**

MicroRNAs (*miRNAs*) are small endogenous non-coding RNAs that control genes at post-transcriptional level. They are essential for development and tissue differentiation, and such altered miRNA expression patterns are linked to the pathogenesis of inflammation and cancer. There is evidence that miRNA expression is genetically controlled similar to the transcription of protein-coding genes and previous studies identified quantitative trait loci (QTL) for miRNA expression in the liver. So far, little attention has been paid to miRNA expression in the skin. Moreover, epistatic control of miRNA expression remains unknown. In this study, we characterize genetic regulation of cutaneous miRNA and their correlation with skin inflammation using a previously established murine autoimmune-prone advanced intercross line.

**Results:**

We identified *in silico* 42 eQTL controlling the expression of 38 cutaneous miRNAs and furthermore found two chromosomal hot-spots on chromosomes 2 and 8 that control the expression of multiple miRNAs. Moreover, for 8 miRNAs an interacting effect from pairs of SNPs was observed. Combining the constraints on genes from the statistical interaction of their loci and further using curated protein interaction networks, the number of candidate genes for association of miRNAs was reduced to a set of several genes. A cluster analysis identified miR-379 and miR-223 to be associated with EBA severity/onset, where miR-379 was observed to be associated to loci on chromosome 6.

**Conclusion:**

The murine advanced intercross line allowed us to identify the genetic loci regulating multiple miRNA in skin. The recurrence of trans-eQTL and epistasis suggest that cutaneous miRNAs are regulated by yet an unexplored complex gene networks. Further, using co-expression analysis of miRNA expression levels we showed that multiple miRNA contribute to multiple pathways that might be involved in pathogenesis of autoimmune skin blistering disease. Specifically, we provide evidence that miRNA such as miR-223 and miR-379 may play critical role in disease progression and severity.

**Electronic supplementary material:**

The online version of this article (doi:10.1186/s12864-016-2455-2) contains supplementary material, which is available to authorized users.

## Background

The discovery of microRNAs (miRNAs), a class of small endogenous non-coding molecules ranging from 18–24 nt brought a new level of complexity for understanding the mechanisms that constitute various biological processes [1, 2]. The involvement of miRNAs in the control of gene expression has been thoroughly defined for the cell cycle, metabolism, and immune system and in cancer [3–6]. Binding to the 3′ or 5′ UTR region of genes, miRNAs may yield increased or decreased gene expression levels and have been described to affect various molecular pathways [7].

Approximately half the miRNAs are intergenic with few also located in intronic regions [8, 9]. These are understood to have their own enhancers and promoters and are transcribed by RNA polymerase II [10]. However, it remains unclear whether these are produced as by-products of protein-coding gene transcription or whether their biogenesis has its own machinery [11]. Recent studies in murine and human fibroblasts of liver tissues revealed that miRNA expression can either be regulated by their transcriptional genomic location or by other regions in the genome [12]. Furthermore, mutations in genes involved in miRNA processing, such as *AGO1*, *DGCR8*, and *DICER*, can cause significant changes in the expression of miRNAs, resulting in altered disease susceptibility [13]. Regarding the skin, in vivo studies show that miRNA biogenesis is dependent on both *DICER* and *DGCR8*. A lack of these enzymes causes severe phenotypes [14], underscoring the importance of miRNAs in the regulation of morphogenesis and homeostasis of the skin [15]. Differentially expressed miRNAs are associated with different physiological and pathological processes in the skin such as melanoma, Sézary syndrome, psoriasis and atopic dermatitis [16–19]. Advancements have been achieved in understanding various processes regulated by miRNAs. On the other hand, the mechanisms that regulate their own expression have so far remained unknown. One possible approach towards understanding their regulation is constraining on genetic loci whose variations statistically link the variable phenotypic effect. We thus performed an *in silico* expression QTL-based analysis, which has been widely used for deciphering genetic loci regulating gene expression in various biological processes [20]. Su et al. have further shown that expression QTL-based analysis can provide insights into miRNA regulation [12]. However, no study has yet been performed to determine the impact of genetic variation on miRNAs associated to skin and autoimmune disorders.

This study (i) uses expression QTL-based analysis to define genetic loci that control miRNA expression in the skin and (ii) provides insights into interacting genes to control a single transcript (epistasis). The relevance of these findings is illustrated by employing a mouse model for the autoimmune blistering disease EBA (Epidermolysis Bullosa Acquisita), providing a direct link between miRNA expression and disease phenotype. Taken together, the data provide insights into the complexity of miRNA regulation and possible means to understand various gene interactions altering the expression of miRNAs.

## Results

A murine heterogeneous inter-cross line was generated by inter-crossing four parental inbred mouse strains: MRL/MpJ, NZM2410/J and BXD2/TyJ as autoimmunity-prone strains and Cast/EiJ for genetic heterogeneity. As a mouse model for an autoimmune blistering disease, 100 mice from generation 4 of the intercross-line were immunized with recombinant collagen type VII (COL7), an integral component of anchoring fibrils located at the dermal-epidermal junction. This induced a loss of tolerance and production of anti-COL7 autoantibodies in all immunized mice. The incidence of sub-epidermal blisters and the clinical phenotype of epidermolysis bullosa acquisita (EBA) were at 1/3 [21]. All mice were genotyped and miRNA expression profiling was acquired from skin tissue using the Affymetrix’s GeneChip miRNA 2.0 array. To reveal genetic loci that regulate miRNA expression, we then performed an association study between the genotype and the respective expression levels of miRNAs. Further we performed co-expression analysis between expression levels of miRNAs and phenotype. The workflow is presented in a flowchart (Additional file 1: Figure S1).

### miRNA expression is genetically controlled

**Fig. 1:**
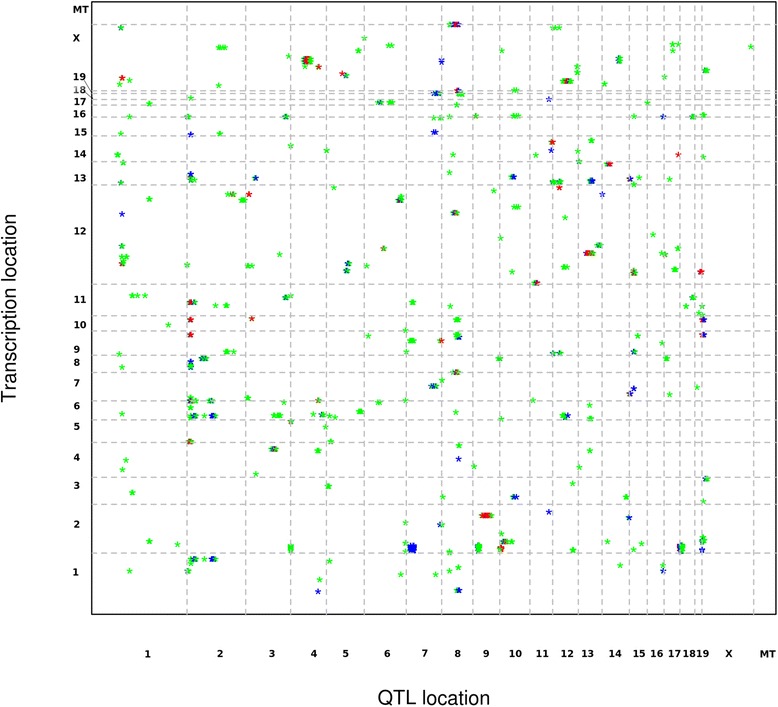
Transcriptome map for eQTL of miRNA . The x-axis in the figure represents the SNP location in the chromosome, and the y axis represents the transcriptional site of miRNAs. In this figure genome wide significant threshold of α < 0.25 is represented in a green. 83 miRNA eQTL were defined as suggestive eQTL for miRNA (genome wide threshold, α < 0.1) are represented in blue. 43 miRNA eQTL with α < 0.05 were observed as significant eQTL are represented in red

We performed a genome-wide scan to detect genetic loci associated with miRNA expression. Expression levels of miRNA were treated as quantitative trait to yield expression quantitative trait loci (eQTL). Genome-wide significance was determined by a permutation test. Using an E-value cutoff of <0.05, 42 eQTL for 38 miRNAs were mapped to the genome, corresponding to 6.83 % of all murine miRNAs present on the above mentioned Affymetrix GeneChip (Table 1 and Fig. 1). Since the wild derived strain CAST/EiJ was incorporated into the advanced intercross line, we investigated the polymorphic sites located in the transcribed miRNAs that may have an effect of probe hybridization which is derived from C57B6/J [22]. We obtained the SNPs and indels in genome of CAST/EiJ from the database [23]. We found that 4/38 (10.53 %) miRNAs (miR-291a-3p, miR-341, miR-449b and miR-681) exhibits indels in CAST/EiJ strain on chromosome 7 (3.2 Mb), 12 (69 and 109 Mb) and 13 (113 Mb), which may suggest false positive associations for those loci (Table 1). The highest -log *P* value of 6.57 was observed for miR-298 on chromosome 9 explaining 20.76 % of the variance. The peak SNP (rs3700596) was found within 1 kb of the *Ube3D* gene, an ubiquitin-conjugating enzyme *E2c* binding protein. We found only trans – eQTL, except for miR-486 (−log *P* = 4.10, rs13479880), which was mapped to the same chromosome of its transcriptional site (Chr 8, ~89 Mb). This indicates that other genes rather than their own transcript may contribute to the regulation of miRNA expression which could be tissue specific.

**Table 1:** eQTL detected for the expression of miRNA

AffyID	PEAK SNP	Chr	Pos(Mb)	CI	-logP score	α	Transcript Location (Chr:Pos)
miR-322	rs6386920	1	54.1	51.5–58.4	4.797697778	0.02	X:50407432-50407526
miR-431	rs6386920	1	54.1	51.5–59.8	5.231417376	0.006	12:110828657-110828747
miR-26a	rs6265423	2	47	28.1–51.3	4.376166299	0.021	10:126432586–126432669,9:118940914-118941003
miR-291a-3p	CEL.2_50605053	2	50.5	28.1–59.9	4.546953451	0.018	7:3218920-3219001
miR-423-3p	rs6265423	2	47	33.9–51.3	4.948513523	0.015	11:76891566-76891674
miR-671-5p	rs13476472	2	45.5	33.9–50.5	5.838570402	0.004	5:24097932-24098029
miR-23b	rs6250599	2	48.5	44.3–51.3	4.335454904	0.022	13:63401792-63401865
miR-409-5p	rs13476874	2	159.5	136–163	4.175101842	0.035	12:110981368-110981446
miR-409-5p	rs13477083	3	43.3	27.5–45.4	4.215972522	0.033	12:110981368-110981446
miR-546	rs13477126	3	56.4	52.9–58.3	4.493016911	0.029	10:126435496-126435616
miR-200a-star	rs3671119	3	126.1	117.4–131.3	4.688008826	0.011	4:155429005-155429094
miR-339-5p	rs3660863	4	7.1	3.7–19.5	4.878132165	0.003	5:139845604-139845699
miR-465c-5p	rs13477873	4	101.1	82.8–118	4.472478928	0.01	X:64079130-64079210
miR-295	rs3663950	4	135.3	129.4–141.1	4.209508065	0.04	7:3220774-3220842
miR-878-3p	rs13478002	4	136.2	135.3–141.1	5.110152979	0.01	X:64054683-64054760
miR-742	rs3673049	5	90.1	87.5–96.6	4.765858909	0.035	X:64033548-64033612
miR-379	rs6208251	6	104.8	98.4–116.7	4.024954772	0.034	12:110947270-110947335
miR-154	rs13479063	6	136.3	133.92–142.4	4.309450382	0.032	12:110976643-110976708
miR-425*	rs3663988	7	146.5	140.2–146.5	4.342220512	0.035	9:108471108-108471192
miR-486	rs13479880	8	89.3	72.5–95.1	4.108040959	0.033	8:24253027-24253154
miR-487b	rs6257357	8	88	77.7–90.2	4.81779528	0.019	12:110965543-110965624
miR-501-3p	rs13479880	8	89.3	81.7–95	4.205948891	0.03	X:6818369-6818477
miR-130b	rs6413270	9	37.7	36.8–44.4	4.222559895	0.032	16:17124154-17124235
miR-298	rs3700596	9	86.2	85–90.6	6.573678024	0.001	2:174093005-174093086
miR-466c-3p	rs3712394	10	17.7	14.1–24.2	4.569565941	0.03	2:10403161-10403244
miR-466c-3p	rs13480563	10	27.9	24.2–38.7	4.591526295	0.029	2:10403161-10403244
miR-126-5p	rs6374078	10	60.6	30.7–65.9	4.177297455	0.031	2:26446877-26446949
miR-681	rs13481076	11	66.5	40.1–71.3	4.055742697	0.043	12:70864822-70864931
miR-20a-star	rs3712881	11	120.9	112–121	4.160612966	0.03	14:115443379-115443485
miR-203	mCV22351241	12	60	55.0–72.6	3.838027866	0.039	12:113369091-113369166
miR-542-3p	CEL.12_84750094	12	91.4	79.7–103.8	4.735149963	0.023	X:50402580-50402664
miR-341	gnf13.079.671	13	80.5	69.5–88.3	5.260091403	0.008	12:110849710-110849805
miR-449b	rs13482231	14	67.6	50.9–72.3	4.485232377	0.019	13:113827627-113827706
miR-7a	CEL.15_4222769	15	4.4	3.2–9.7	4.941341377	0.044	13:58494140–58494247, 7:86033163-86033259
miR-7a	rs13482455	15	16.7	14.8–24.5	5.73214998	0.022	13:58494140–58494247, 7:86033163-86033259
miR-337-3p	rs13482549	15	45.5	38.4–53.9	4.298224443	0.029	12:110823999-110824095
miR-673-5p	rs13482549	15	45.5	38.9–56.6	4.592339382	0.014	12:110810200-110810290
miR-136	rs13482914	17	21	16.5–27.6	4.679083445	0.026	12:110833537-110833598
miR-466b-5p	rs13483212	18	12.3	0–21	4.48515896	0.016	2:10395846-10395927
miR-493	rs6211533	19	57.1	50.2–60.2	4.98913787	0.004	12:110818443-110818525
miR-26a	gnfX.023.543	X	36.3	0–49.3	4.718906745	0.01	10:126432586–126432669, 9:118940914-118941003
miR-466e-5p	rs13483712	X	9.1	0–33.5	4.013512497	0.05	2:10398257-10398340

*AffyID* Affymentrix ID, *Chr* Chromosome, *Pos (Mb)* Peak Position SNP in Mb, *CI* Confidence Interval (CI) in Mb using 1.5 -log P drop, *α* Genome wide significance

Prior knowledge suggests classes of gene families that play a major role in the regulation of miRNA expression i.e. Argonaute proteins and helicases which play a crucial role in its regulation [24]. To examine this assumption, we obtained the coordinates of all helicases and other genes involved in pathways of miRNA biogenesis from ‘The RNA Helicase Database’ and mapped them to the eQTL identified in our study [25]. Helicases like *Ddx39*, *Ddx49*, *Cd97* and *Upf1* were mapped within the confidence interval of miR-486, miR-487b and miR-501 on chromosome 8. Further, four helicases (*Ddx50*, *Ascc3*, *Ddx21* and *Dna2*) were mapped within the confidence interval of the eQTL controlling miR-126. Genes that are involved in transcriptional processes, such as *Polr3f*, *Polr2a*, *Polr3g* and *Polr2a* were also mapped to the eQTL for miR-409, miR-681, miR-34 and miR-449. Other genes which do not belong to these classes, such as lin28a and its homolog lin28b, have been shown to modulate let-7a [26]. These genes were mapped to the eQTL for miR-290 on chromosome 4 and miR-126 on chromosome 10, suggesting that *Lin28* might modulate the expression of other miRNAs as well.

**Fig. 2:**
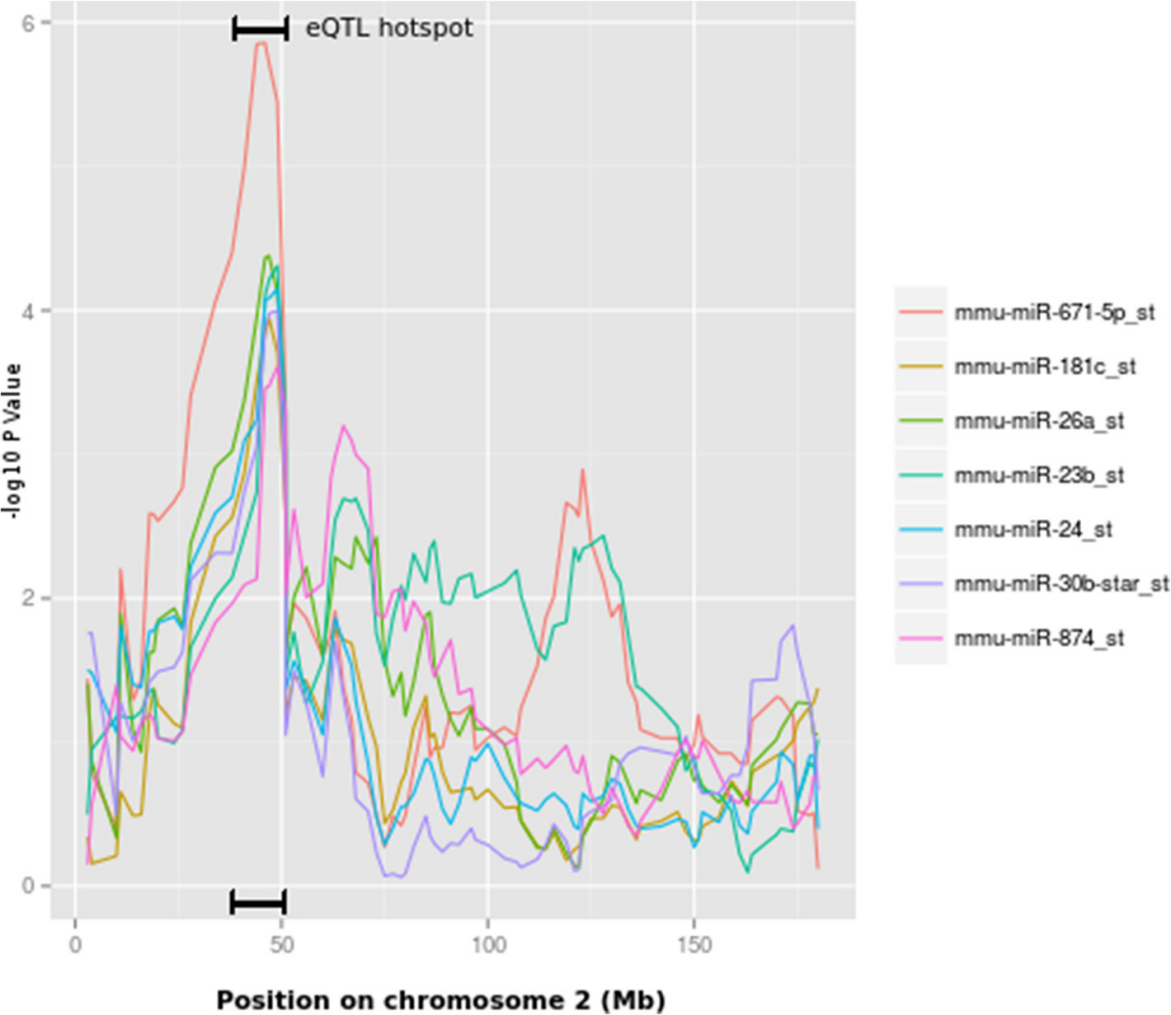
eQTL hot spots on chromosome 2. Position on x axis represents the coordinates in million base pairs on chromosome 2. The y axis is –log *P* value. The overlapping peaks on y axis represents an eQTL hot spot between 28–51 Mb. miR-181, miR-30b* and miR-874 are additional suggestive eQTL with significant genome-wide (α < 0.1) threshold after permutation

Some miRNA eQTL were constrained to a specific location in the genome, thereby suggesting potential eQTL hotspots for the regulation of multiple miRNAs. On chromosome 2, five miRNAs (miR-26a, mir-291a, miR-423, miR-671 and miR-23b) were mapped between 28–51 Mb (Fig. 2). Three nearby SNPs (rs6250599 ~ 48.5 Mb, rs6265423 ~ 47 Mb and rs13476472 ~ 45 Mb) showed significant association (−log *P* > 4) with all five miRNAs that were mapped to this region. A long non coding RNA *1700019E08Rik* was located near SNP rs13476472 (~3 Kb upstream). As for the other two SNPs, the nearest gene to rs6265423 was mapped 6.8 kb apart, coding for the snRNA *U7.39-201*, while the nearest coding gene for SNP rs6250599 was a pseudogene *Gm13489-001* (~13 kb upstream).

Similarly, on chromosome 8 we identified eQTL for three miRNAs (miR-486, miR487b and miR-501) in confidence interval 72–95 Mb. Three genes *Gm1068*, *Siah1* and *Dnaja2* were located near peak SNPs (rs13479880 and rs6257357).

Furthermore, we found several miRNAs that are associated to more than one locus in the genome. For example miR-7a showed significant association with two loci present on chromosome 15 (3–9 Mb, CEL.15_4222769 and 14–24 Mb, rs13482455). Similarly, miR-466-3c was regulated by two nearby loci on chromosome 10 (14–24 Mb, rs3712394 and 24–38 Mb, rs13480563). Additionally, miR-26a was mapped with two loci on chromosomes 2 and X (28–51 Mb, rs6265423 and 0–49 Mb, gnfX.023.543).

### Epistatic control of miRNA expression

**Fig. 3:**
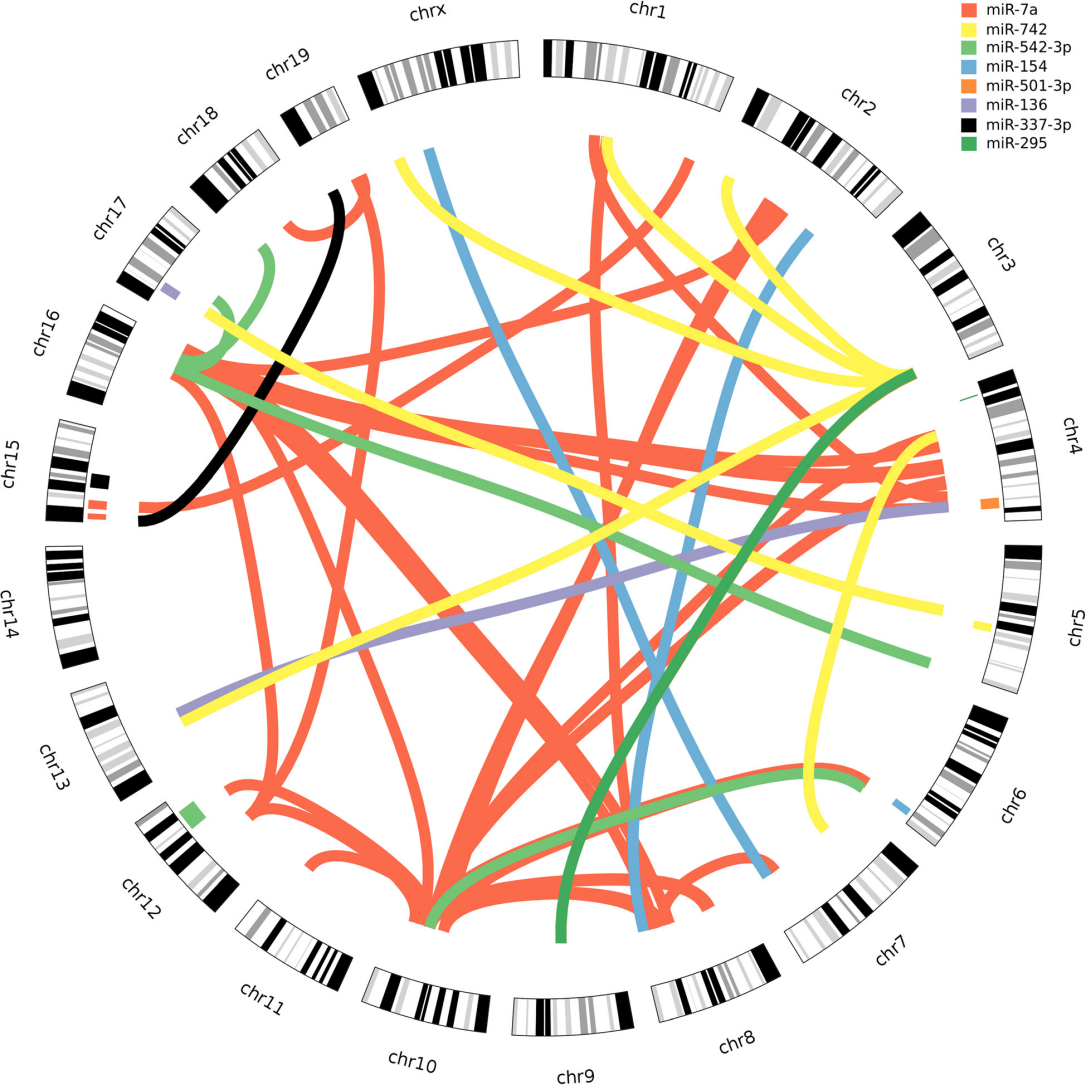
Epistasis in miRNA eQTL. The circular plot shows the chromosomes in their circumference. Each connecting line represents a SNP pair interaction above the significance level (adj. *P* < 0.05). Each interaction is color coded for different miRNAs. The boxes adjacent to the chromosomal band show eQTL for miRNAs mapped for single locus scans

A defective of single loci may possibly be compensated by another, or only jointly the effect is the strongest. Therefore, for all the miRNAs significant single-locus eQTL, we analyzed the epistatic effect for each SNP pair. As a result, we identified 200 SNP pairs for 8 miRNAs below the significance level (adjusted *P* value < 0.05) (Fig. 3 and Additional file 1: Table S1). The highest -log *P* value of 10.38 was found between the SNP pairs rs13480360 (chr 10, ~67 Mb, nearest gene: *AK139516*) and rs3689658 (chr 2, ~ 85 Mb, nearest gene: *Olfr1006*) for miR-7a (Table 2). In total, we found 119 SNP pairs for miRNA miR-7a. The hub locus (i.e. SNP with the maximal number of interactions) for miR-7a was observed on chromosome 16 (rs3680665 ~ 84 Mb, nearest gene: *AK04263*). The same SNP (rs3680665) also showed association with miR-542 additionally with another SNP (rs4200124, nearest gene: *Gbe1*) present nearby. For miR-742 we observed 47 SNP pairs with SNP rs3657112 (~148 Mb, nearest gene: *Snora17*) on chromosome 3 showing the highest number of associations (40 SNP pairs). The same SNP i.e. rs3657112 also showed statistical interaction with chromosome 9 loci 67–72 Mb for miR-295. We also found multiple SNPs on chromosome 2 (13–17 Mb) statistical interacting with SNPs on chromosome 1(3–11 Mb) for miR-501. Two miRNAs (miR-136 and miR-337) had only one significant SNP pair: for miR-136 the correspondence was found between SNPs rs37113033 (chr 19, ~5 Mb, nearest gene: *Slc29a2*) and rs13459176 (chr 15, ~3 Mb, nearest gene: *Sepp1*), while miR-337 had SNP pair rs3693942 (chr 13, ~55 Mb, nearest gene: *Unc5A*) and rs3663950 (chr4, ~135 Mb, nearest gene: *Il22ra*) (Table 2).

**Table 2:** Epistasis in miRNA. The table includes only the top interacting SNPs for each miRNAs

miRNA	Top interacting SNP 1					Top interacting SNP 2					I.Pval(log)
	SNP ID	chr	Pos(Mb)	Refseq Genes	Pval(log)	SNP ID	chr	pos(Mb)	Refseq Genes	Pval(log)	
miR-7a	rs13480630	10	67.29		0.89	rs3689658	2	85.52	Olfr1006	1.12	10.4
miR-136	rs3713033	19	5.03	Slc29a2	0.073	rs13459176	15	3.23	Ccdc152, Sepp1	1.56	7.2
miR-154	rs3708073	8	124.28	Gm20388,Jph3	0.3	rs8246404	2	136.7	Mkks	0.57	7.67
miR-295	rs13480271	9	72.68	RP23-461P14	0.018	rs3657112	3	148.03		0.32	8.064
miR-337-3p	rs3693942	13	55.05	Unc5a	0.026	rs3663950	4	135.29	Il22ra1	0.2	7.17
miR-501-3p	CZECH-2_15618849	2	15.5	Gm13364	1.33	rs3716083	1	9.01	Sntg1	0.72	8.46
miR-542-3p	rs4200124	16	70.7		1.12	rs3718776	5	150.4	Wdr95	0.14	8.5
miR-742	rs3657112	3	148.028		0.35	CEL.1_49993068	1	49.68		0.33	9.16

*Chr* Chromosome, *Pos (Mb)* Position in million base pairs, *Pval(log)* Additive log 10 *P* value, *I.Pval(log)* Interacting log 10 *P* value

To search the candidate interacting gene pairs we investigated the epistatic control of miR-501-3p. Using the Ingenuity pathway analysis (IPA), we searched for all the possible interacting genes present between loci present on chromosome 1 and chromosome 2 [27]. We found three putative interacting gene pairs; namely *Commd3* with *Cops5*, *Cacnb2* with *Vopip1* and *Commd3-Bmi1* with *Rb1cc*. Interestingly, *Cops5* in the *Nfkb1* pathway possibly be associated with miR-501 via *Tp53* (Additional file 1: Figure S2).

### Genetic overlap of QTL for clinical scores and miRNA expression

**Fig. 4:**
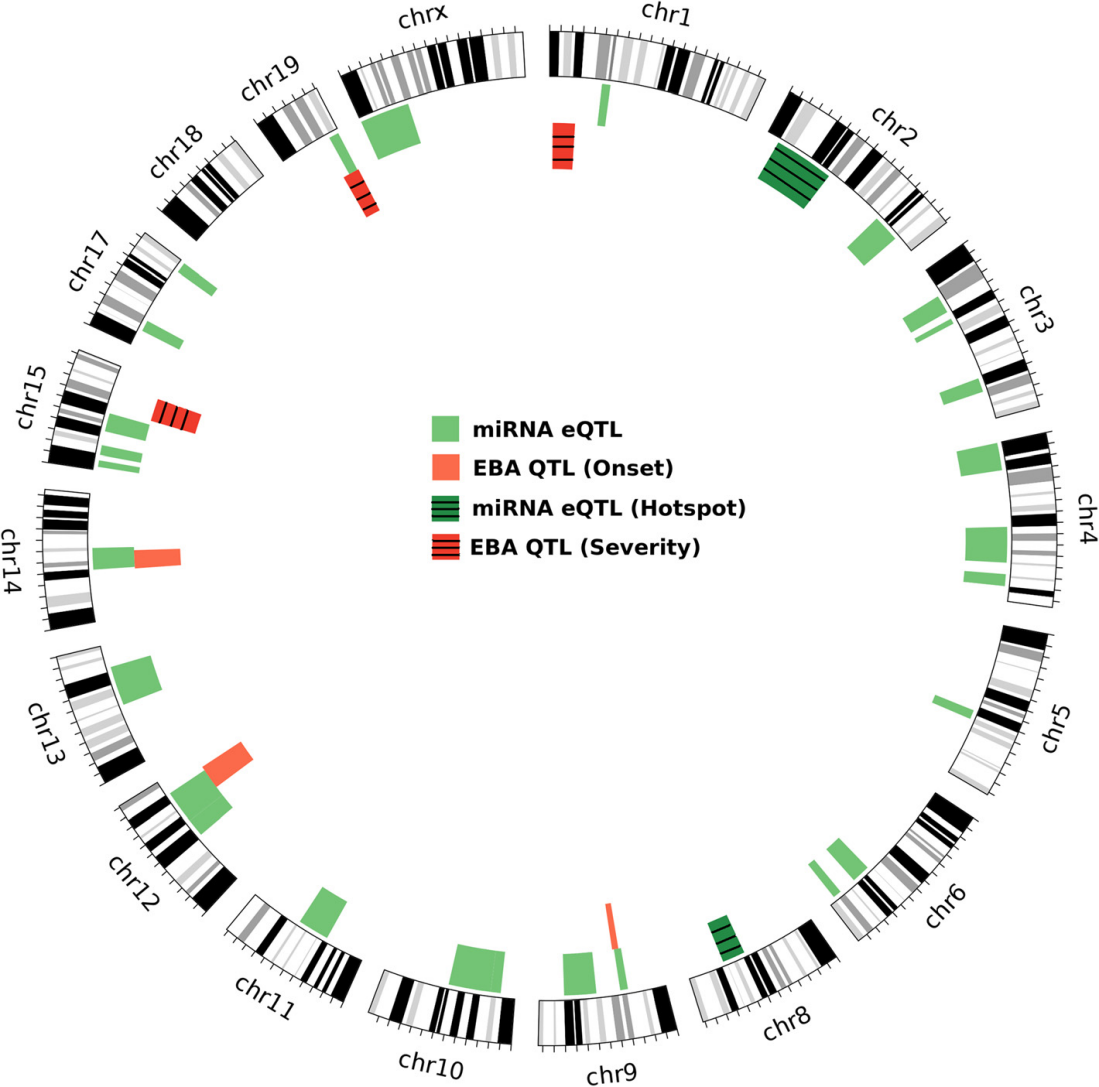
Overlapping QTL for EBA disease and miRNA eQTL. The circular plot shows all the eQTL for miRNAs (green) and QTL for EBA (red). It also presents the eQTL hot spots (dark green) and EBA QTL for onset (red) and severity (dark red). Each circular band represents a chromosome on which QTL and eQTL are mapped. The region within the chromosome which has either red or dark red and green or dark green bands is overlapping eQTL with EBA QTL

**Fig. 5:**
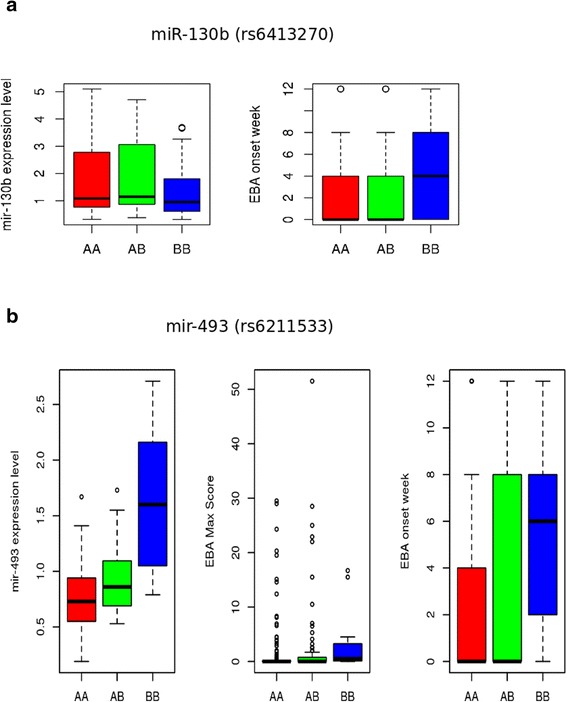
Boxplot for genotype variations for significant SNP in miRNA eQTL for disease QTL. The figure describes the genotype variation for the peak SNP for miRNAs mapped to the previously described EBA QTL. **a**) In miR-130b eQTL, peak SNP (rs6413270, −logP = 4.22) have genotype AA for BxD2 and CAST/EiJ while BB for NZM/2410J and MRL/MpJ strain. The variation associated with three genotypes AA, AB and BB for expression of miR-130b is presented on left and onset week of EBA on right box. **b**) In miR-493 eQTL, peak SNP (rs6211533, −logP = 4.99) have genotype AA for BxD2, CAST/EiJ and NZM/2410J while BB for MRL/MpJ strain. The variation associated with three genotypes AA, AB and BB for expression of miR-493 is on left, maximum score of severity of EBA disease in middle and onset week of EBA on right

Any genetic variation with an effect on the clinical phenotype and miRNA levels should map to the same chromosomal region. Previously, genetic loci for the clinical phenotype of EBA, an autoimmune skin blistering disease, were studied using a larger cohort of mice from the same breeding scheme. Previously, we mapped the QTL for the onset of EBA to chromosomes 9 (39.5–46.3 Mb), 12 (83.2–109.9 Mb), 14 (49.1–68.9 Mb) and 19 (46.0-end Mb). Additionally, three QTL were mapped for severity of EBA on chromosomes 1 (3.1–27.3 Mb), 15 (61.0–85.7 Mb) and 19 (43.1-end Mb) [21]. In this study, we found 4 eQTL for miRNAs overlapping with the QTL for EBA (Fig. 4). The eQTL for miR130b (Chr 9: 36–44 Mb, −log *P* = 4.42), miR-542-3p (Chr 12: 79–103 Mb, −log *P* = 4.73) and miR-449b (Chr 14: 50–72 Mb, −log *P* = 4.49) were mapped on QTL for disease onset on chromosome 9, 12 and 14. Additionally, we mapped eQTL for miR-493 (50–60 Mb, −log *P* = 4.98) to the QTL for both disease severity and onset on chromosome 19. To further evaluate, if the variation in the genome is associated with the miRNA expression levels and also underlies the clinical phenotype, we investigated the genotype association of significant SNPs of miRNA eQTL with clinical phenotype (Fig. 5). We observed that the most significant SNP (rs6211533) mapped for the eQTL of miR-493 on chromosome 19 also shows variation for EBA severity score and onset. In this eQTL minor allele (BB) derived from MRL/MpJ was associated with higher expression of miR-493, late onset of the disease, and higher clinical score (Fig. 5b). Similar observation was also derived for eQTL mapped (peak SNP, rs6413270) for miR-130b where minor allele (BB) derived for MRL/MpJ and NZM/2410J was associated with lower expression of miR-130b, and late onset of disease phenotype (Fig. 5a).

### Expression and co-expression of miRNAs

**Fig. 6:**
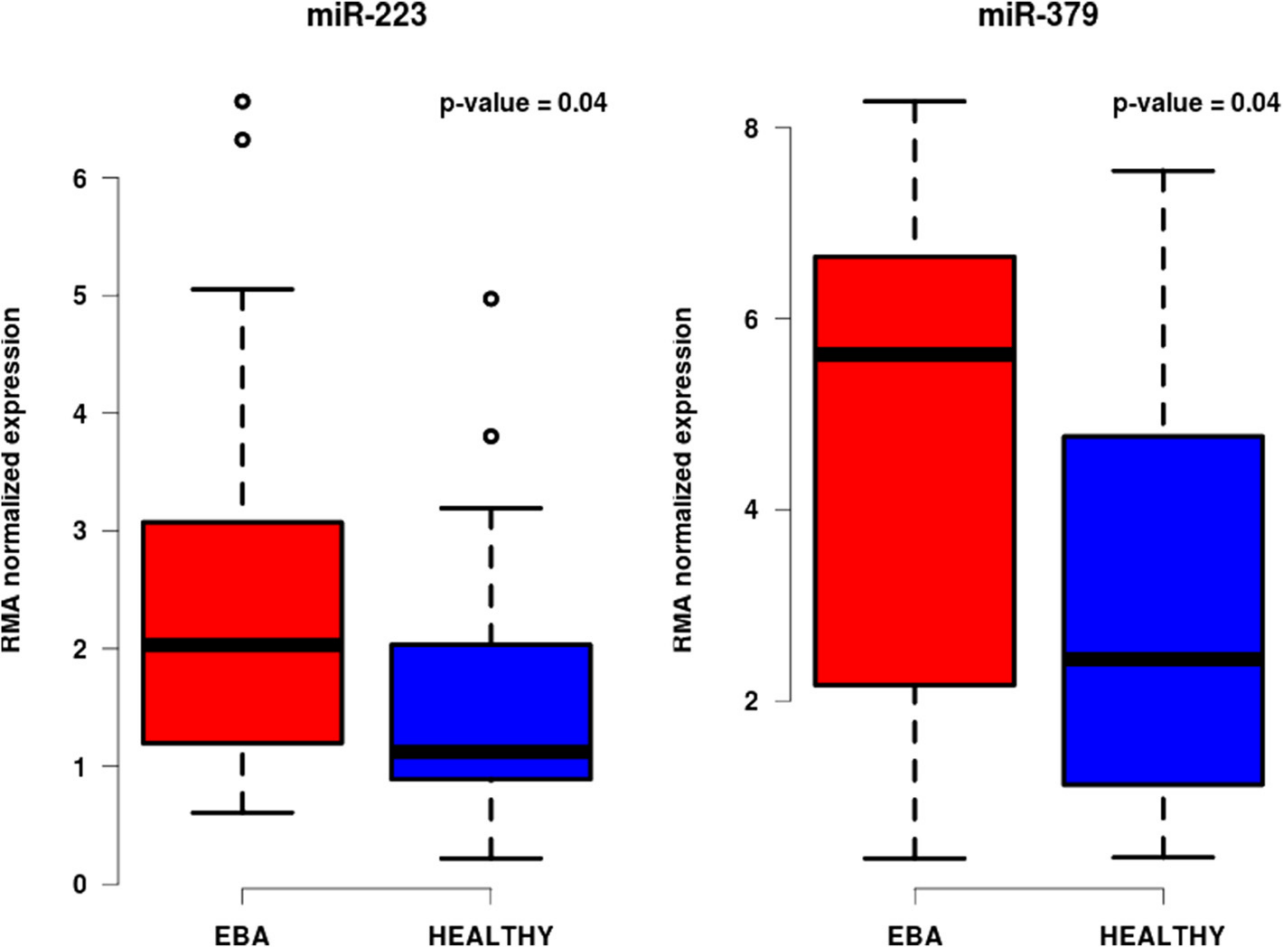
Boxplot showing differentially expressed miRNAs. The box plot shows the most differentially expressed miRNAs miR-223 and miR-379 for the disease phenotype EBA. The plots in blue color show the expression of miRNAs in mice with no clinical phenotype while mice with signs of inflammation are shown in red

Immunization of mice with recombinant COL7 leads to development of subepidermal blisters and the clinical phenotype of EBA in 1/3 of the immunized mice, while 2/3 remain clinically healthy. To access differential expression of miRNAs, we divided the 4^th^ generation of our mouse cohort into two separate groups: affected and non-affected mice. Comparing the two groups, only two miRNAs were differentially expressed: miR-379 (adj. *P* value = 0.044) and miR-223 (adj. *P* value = 0.044) (Fig. 6 and Additional file 1: Table S2). Both miRNAs were significantly over expressed in mice with disease. Additionally, an eQTL for miR-379 (−log *P* = 4.7, 98–116 Mb) was also mapped on chromosome 6, with its peak at ~104 Mb (rs6208251, nearest gene: *Cntn6*).

**Fig. 7:**
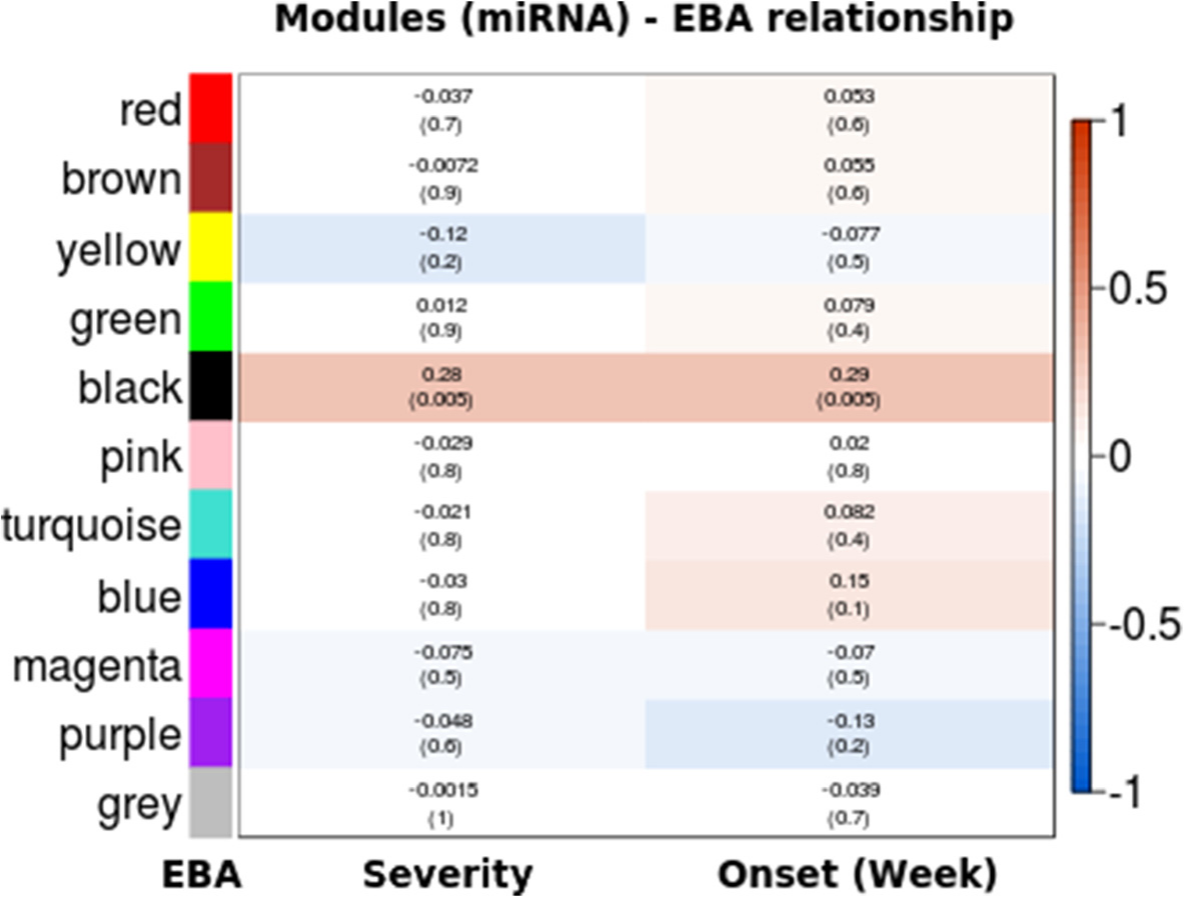
Module-trait relationship between clusters of miRNAs with EBA severity and onset. The graph is a representation of co-expression analyses of miRNAs. The clusters of miRNAs are called modules which are color coded on the y-axis. The disease phenotypes (traits) are EBA onset and EBA severity given on the x-axis. Blocks represent the correlation of module with phenotype using a Pearson correlation coefficient ranging from −1 to 1. The range is color coded with red representing positive correlation and blue representing negative correlation. *P* values are given in brackets below the correlation coefficients

The comparison of diseased and non-diseased mice has its drawbacks; the disease severity observed for individual mice might differ depending on the differences in the genome. To investigate miRNA expression affecting disease severity, we performed co-expression analysis among the expression levels of miRNAs (co-expressed miRNAs are in same pathway) in correlation with quantitative scores for severity and onset week of disease. For this purpose, we employed the WGCNA R package, which clusters miRNAs into different modules and further associates them to a phenotypic score, such as EBA severity or disease onset. As a result, we identified 11 clusters (Fig. 7). Using this approach, only the ‘black’ module, consisting of 23 miRNAs, was significantly associated with EBA (ρ = 0.28, *P* = 0.005) (Additional file 1: Table S3). Additionally, it was significantly correlating to the onset of EBA (ρ = 0.29, *P* = 0.005). Due to the fact that the ‘black’ module stronger correlates with the onset of the disease than with the maximum score, one can speculate that miRNAs from this module is rather involved in the onset than in the severity of the disease. This would relate to our previous observation, in which miRNA eQTL were overlapping with EBA onset QTL. The pathways associated with the ‘black’ module were predominantly pathways which have been shown to play a crucial role in other autoimmune disorders, such as the MAPK signaling pathway, T-cell receptor and TGF-beta (Table 3).r

**Table 3:** List of over-represented KEGG pathways for ‘black’ module

Total Genes of the Term	Union	Targets in the Term	Union miRNAs in the Term	Score
AXON_GUIDANCE	131	51	17	1.88
PATHWAYS_IN_CANCER	323	98	18	1.788
MAPK_SIGNALING_PATHWAY	271	89	17	1.69
NEUROTROPHIN_SIGNALING_PATHWAY	131	45	16	1.557
T_CELL_RECEPTOR_SIGNALING_PATHWAY	109	42	16	1.424
ENDOCYTOSIS	219	65	16	1.408
TGF-BETA_SIGNALING_PATHWAY	85	30	15	1.334
GLIOMA	65	27	16	1.319
PROSTATE_CANCER	89	32	17	1.314
UBIQUITIN_MEDIATED_PROTEOLYSIS	138	52	16	1.277
COLORECTAL_CANCER	65	25	16	1.271
WNT_SIGNALING_PATHWAY	153	38	18	1.268
MELANOGENESIS	100	34	18	1.256
ERBB_SIGNALING_PATHWAY	87	33	17	1.246
MTOR_SIGNALING_PATHWAY	53	22	13	1.154
INSULIN_SIGNALING_PATHWAY	137	45	16	1.151
B_CELL_RECEPTOR_SIGNALING_PATHWAY	76	23	16	1.137
FOCAL_ADHESION	197	50	18	1.111
ADHERENS_JUNCTION	74	28	17	1.097
CHAGAS_DISEASE	102	34	16	1.094
GNRH_SIGNALING_PATHWAY	99	32	15	1.042
MELANOMA	71	26	16	1.037
PANCREATIC_CANCER	70	27	15	1.028
ENDOMETRIAL_CANCER	52	19	16	1.022

Individual correlation of miRNA expression levels with the disease severity identified 24 miRNAs to be significant (*P* < 0.05) (Additional file 1: Table S3), with miR-223 showing the strongest association (ρ = 0.4, *P* = 4.93e-05). To further validate this result we experimentally verified the expression of miR-223 in EBA skin by qRT-PCR. We found that miR-223 is upregulated in EBA skin in comparison to normal skin (Additional file 1: Figure S3). Another miRNA which was highly correlating with EBA was miR-21 (ρ = 0.36, *P* = 0.00036). Further, investigations of the co-expression module showed that miRNAs were potentially co-regulated i.e. possible overlapping genetic loci among co-expressed miRNAs. We observed that miRNAs in a given locus were either clustered within the same module or showed stronger inter-module membership for a specific module even if they were assigned to different modules. As an example, miR-322 and miR-431, that were mapped on chromosome 1 (51–59 Mb), are clustered in the ‘red’ module. In eQTL hot spots as on chromosome 2 (28–51 Mb), miR-423-3p and miR-23b are clustered in the ‘yellow’ module. Even though other miRNAs in this locus were assigned to a different module, they also show significance for the ‘yellow’ module. Examples are given by miR-671-5p (*P* = 1.224713e-12), miR-26a (*P* = 2.654271e-19) and miR-291a-3p (*P* = 3.530522e-02) (Additional file 1: Table S3). In line with this observation, for the eQTL mapped on chromosome 8, two miRNAs (miR-501-3p and miR-486) were clustered with the ‘brown’ module while miR-487 was assigns to the ‘red’ module but had significant module membership with the brown module as well (*P* = 0.004). Thus, these genomic loci may be considered as confirmed and strengthen the genetic contribution for the control of miRNAs expression levels.

## Discussion

Small non-coding RNAs like miRNAs are known to contribute to the onset and severity of various diseases as well as the defense against them [28]. Thus, miRNAs function as tissue-specific key regulators, affecting some of the major pathways towards an aggravation of disease severity when aberrantly expressed [29]. Accordingly, it is not of surprise that miRNAs have been recently recognized as potential therapeutic targets [30, 31].

However, the underlying mechanisms of such dysregulated miRNA expression patterns are not well characterized. Different studies have shown that gene expression alterations in different tissues are genetically derived [29]. Thus, it is plausible that not only the regulation of gene expression is genetically controlled, but also the expression of miRNAs. In this study we explore the diversity of miRNAs in inflamed skin tissue and genetic loci that control variations in miRNA expression levels across a mouse cohort. We provide evidence that miRNA levels in skin tissue are genetically controlled on transcriptional level by helicases and RNA polymerases that are important for the biogenesis of miRNA. Furthermore, we found that some of the miRNA eQTL are restricted to one particular locus in the genome (eQTL hot spots). Deeper investigation revealed that these miRNAs are under multi-locus and/or epistatic control.

Interestingly, eQTL hot spots were predominantly found in genomic regions coding for non-coding RNA. Hence, it is tempting to speculate miRNA expression might not necessarily be solely controlled by protein-coding RNA, but rather by non-coding RNA which would impose an additional level of post-transcriptional regulation. This in turn leads to the tempting hypothesis that non-coding RNAs do at least in part regulate miRNA expression. Such a scenario is supported by the fact that some non-coding RNAs have been shown to bind to miRNAs at functional level as demonstrated by an interaction of *linc-MD1* with miR-133 and miR-135 [32]. Here, *linc-MD1* works as a sponge and traps these miRNAs preventing the binding to the canonical targets. Moreover, a recent study even shows an interaction network between lncRNAs and miRNAs [32].

Based on our observed overlap between QTL controlling miRNA expression and EBA, a blistering phenotype in autoimmune skin disease, we conclude that there are interconnected pathways to simultaneously regulate both disease development and miRNA expression. This might explain the findings of earlier studies that show a clear correlation of aberrant miRNA expression and autoimmune diseases [33–36]. Accordingly, initiation and/or progression of the disease do not primarily appear to be caused by aberrant miRNA expression. Quite the contrary seems to be true; aberrant miRNA expression could be a consequence of the disease which in turn would lead to a downward spiral. Hence, miRNAs could provide a large and unexplored reservoir of potential biomarkers for EBA and related cutaneous autoimmune skin blistering diseases and an interesting target for therapeutic intervention.

## Conclusion

Taken together, our study provides a complex framework of gene-gene and miRNA-gene-interactions, which eventually leads to disease development and progression. Our data provide evidence that miRNAs are important drivers of cutaneous autoimmune diseases by acting on various pathways. Moreover, the study strongly implies there is yet another, so far largely unexplored level of regulatory network, possibly comprised of by non-coding RNAs which on their part affect miRNA expression. In this sense, aberrant miRNA expression would indeed be one the responsible elements for disease progression, however, the driving force behind might be a different one.

## Methods

### Generation of a 4-way advanced intercross line

The out bred four-way autoimmune-prone advanced intercross line (AIL) was generated (in our group) from the parental mouse strains BXD2/TyJ, MRL/MpJ, NZM2410/J and CAST/EiJ [21, 37, 38]. All strains were purchased from The Jackson Laboratory (Bar Harbor, ME). The four inbred strains were intercrossed following an equal strain and sex distribution. First generation (G1) offspring mice were then mated based on their parental origin to generate G2 mice in order to maintain an equal distribution of the original strain alleles across the genome. The same procedure was applied for intercrossing G2 and G3 mice. At least 50 breeding pairs were used for each successive generation of mice. Animals were held under pathogen free conditions at a 12-h light/dark cycle with food and water ad libitum. All animal experiments were approved by the Ministerium für Energiewende, Landwirtschaft, Umwelt und ländliche Räume des Landes Schleswig Holstein in Kiel, Germany (Refrence number : V 312–72241. 122–5 (12-2/09)).

### Induction of experimental EBA

Experimental EBA was induced by immunization with an immune-dominant peptide within the murine NC1 domain of Collagen type VII (GST-mCOL7C) as previously described [39]. In total, 600 mice of the AIL were immunized out of which 100 mice were randomly selected for miRNA expression profiling. Mice were evaluated every 4th week after immunization regarding their development and extent of skin disease, following an established scoring system for a total period of 12 weeks [40]. Murine skin tissues were obtained for analysis at the end of the experiment.

### Expression profiling and statistical analysis

Total RNA was extracted from skin tissue and processed as previously described using the Flash Tag Biotin HSR RNA labeling kit and hybridized with the miRNA expression profiling GeneChip miRNA 2.0 Array according to the manufacturer’s protocol [41]. Raw data was pre-processed using R packages and normalized using RMA (Robust Multi-array analysis) to generate expression levels across the samples following instruction of at bioconductors.org [42].

### Genotyping and expression QTL analysis

Genomic DNA was isolated from tail clippings and incubated in 500 μl 50 mM NaOH at 95 °C for 2 h. The reaction was neutralized by posterior addition of 50 μl 1 M Tris HCl (pH 8.0). DNA was further processed with DNeasy Blood & Tissue Kit according to manufacturer’s instructions. The extracted DNA was quantified using Nanodrop and normalized to 50 ng/μl in TE buffer (10 mM Tris; 1 mM EDTA; pH = 8). Agarose gel electrophoresis was performed for quality control. A total of 1400 SNPs markers evenly spaced across 19 autosomes and the X chromosome were genotyped on 100 generation 4^th^ mice using Illumina mouse medium density array. 200 SNPs were discarded as they were non informative and 1200 markers were retained. The marker order and position in our map is provided in (Additional file 1). The SNP genotype information for each mouse from generation 4 and four founders is provided in (Additional file 1). These informative SNPs were used for performing eQTL analysis using HAPPY 2.3 on Debian Linux for miRNA expression levels [43, 44]. The software infers the haplotype probabilities for each sample using the progenitor SNP information. The miRNA expression levels of mice (600 probes) were considered as quantitative traits and linkage analysis with haplotype probabilities was done using the additive gaussian model. We considered gender as additive covariate. 1000 permutations were performed across all miRNA probes. A significant threshold (α = 0.05) was defined genome-wide. Correction for family structure was done using a variance component model: A Kinship matrix was obtained by the EMMA R package to account for relatedness among the individual [45] and interpreted as a random effect with sex as fixed effect to perform single marker association with phenotype.

We defined the confidence interval of the given eQTL based on a -log *P* value drop of 1.5. We considered local regulation if the eQTL for the probe was found on the same chromosome as its genomic location. A trans-regulation was presumed if the QTL controlling the trait was found in a different chromosome than its genomic location. The function “epistasis” from the HAPPY R package was used to find pair wise SNP interactions using F-score for the interaction model for SNPs which were significantly associated with miRNA expression levels in initial genomic scan [43]. All the *P*-values are corrected for multiple testing using the Bonferroni correction. Circos was used for visualization of eQTL and epistasis [46].

### Co-expression analysis

The standard WGCNA procedure was used for module detection [47]. For miRNA we considered 97 samples. 3 samples were excluded as these could not phenotype for EBA score. A weighted adjacency matrix of pair-wise connection strengths (correlation coefficients of gene expression levels) was constructed using the soft-threshold approach with a scale independent topological power β = 7 (miRNA). For each probe, the connectivity was defined as the sum of all connection strengths with all others. Probes were aggregated into modules by hierarchical clustering and refined by the dynamic cut tree algorithm [48]. The Pearson correlation coefficient was determined for each phenotype-module pair. The representative module expression profile, or module eigengene value, is the first principal component of the gene expression profile within a module. The correlation between the module eigengene and the sample trait of interest yields the eigengene significance, as assessed by a correlation test. The modules were assigned by different colors where grey was assigned to traits that could not be clustered in any other module. To determine the enriched pathways involved in a cluster we used ‘miRsystem’ which uses the weighted pathway-ranking method for identifying enriched biological functions [49].

### Total RNA preparation, cDNA synthesis and qRT-PCR

Perilesional skin samples from mice injected with NC1 domain of Collagen type VII (GST-mCOL7C) or normal skin from mice injected with GST alone were obtained. Total RNA was extracted using TRIzol® reagent (Invitrogen GmbH, Darmstadt, Germany). RNA concentrations were measured on a Nanodrop 2000c spectrophotometer (Thermo Fischer Scientific GmbH, Bremen, Germany). Total RNA was used for cDNA synthesis as previously described [50]. Briefly, 100 ng of total RNA were poly (A) tailed and reverse transcribed in a single reaction tube containing: 1 μl of 10xpoly(A) polymerase buffer, 0.1 mM of ATP, 0.1 mM of dNTPs, 100 units of MuLV reverse transcriptase (New England Biolabs, Frankfurt am Main, Germany), 1 unit of poly(A) polymerase (New England Biolabs, Frankfurt am Main, Germany), and 1 μM of RT primer (5′-CAGGTCCAGTTTTTTTTTTTTTTTGT-3′) in a final volume of 10 μl. Consequently, the tube was incubated at 42 °C for 1 h followed by 5 min at 95 °C. The cDNA was diluted 1:10 before the qPCR reaction. qPCR analysis was performed on a Mastercycler ep Realplex (Eppendorf AG, Hamburg, Germany) using 8 μl of diluted cDNA, 10 μl of SYBR Select Master Mix (Thermo Fischer Scientific GmbH, Bremen, Germany), and 250nM mmu-mir-223 specific primer set. The cycling conditions were 50 °C for 2 min, 95° for 2 min, followed by 40 cycles of 95 °C 15 s and 60 °C for 1 min. The expression levels of mmu-mir-223 were normalized against β-actin. The sequences of the qPCR primers used in this study are:mmu-mir-223 F: 5′-CGCAGTGTCAGTTTGTCA-3′; mmu-mir-223 R:5′-CCAGTTTTTTTTTTTTTTTGGGGTA-3′; mmu-β-actin F:5-CCCCATTGAACATGGCATTG-3′; mmu-β-actin R: 5′- ACGACCAGAGGCATACAGG-3′.

## Availability of the supporting data

Data is accessible at NCBI GEO GSE64276.

## Additional file

Additional file 1: Figure S1. Flowchart describing the workflow of analysis. The flowchart provides an overview of the analysis performed for understanding regulation of miRNAs and their contribution to the disease phenotype. **Figure S2** Interaction network accessed via IPA software for epistasis of miR-501. The graph depicts the interacting genes identified from epistasis scan of miRNA miR-501 in chromosome 1 and chromosome 2. The graph shows all known gene interactions between the two loci where genes colored in yellow are from locus on chromosome 2 and green are from locus on chromosome 1. The red line shows the possible pathway for the regulation of miR-501. **Figure S3** qRT-PCR validation of miR-223 expression in EBA and normal murine skin. (ZIP 637 kb)

## Abbreviations

EBA: epidermolysis bullosa acquisita
eQTL: Expression quantitative trait loci/locus
lncRNA: Long non coding RNA
miRNA: Micro RNA
QTL: Quantitative trait loci/locus
SnoRNA: Small nucleolar RNA
SnRNA: Small nuclear RNA
