# Supplementary figures and images for: Dissecting genetics of cutaneous miRNA in a mouse model of an autoimmune blistering disease
Source: BMC Genomics. 2016 Feb 16;17:112. doi: 10.1186/s12864-016-2455-2 (PMC4755013; doi:10.1186/s12864-016-2455-2)

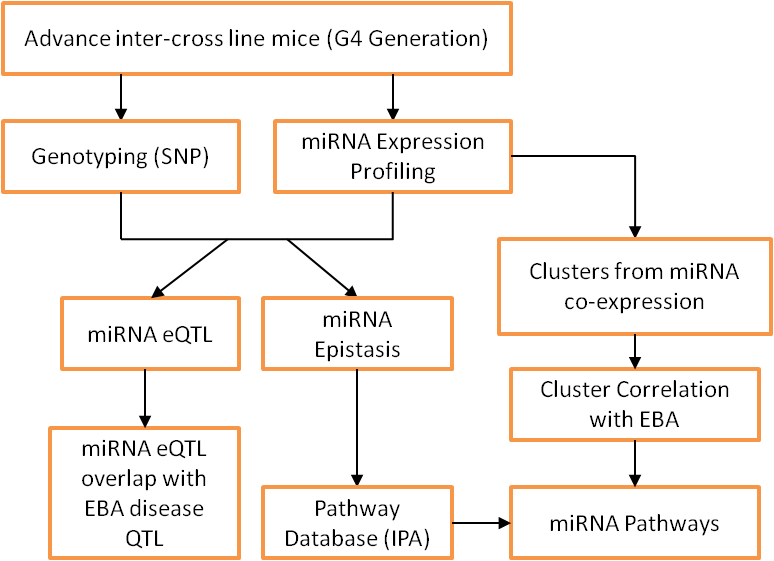

Supplement: Additional file 1: Figure S1. — Flowchart describing the workflow of analysis. The flowchart provides an overview of the analysis performed for understanding regulation of miRNAs and their contribution to the disease phenotype. Figure S2 Interaction network accessed via IPA software for epistasis of miR-501. The graph depicts the interacting genes identified from epistasis scan of miRNA miR-501 in chromosome 1 and chromosome 2. The graph shows all known gene interactions between the two loci where genes colored in yellow are from locus on chromosome 2 and green are from locus on chromosome 1. The red line shows the possible pathway for the regulation of miR-501. Figure S3 qRT-PCR validation of miR-223 expression in EBA and normal murine skin. (ZIP 637 kb) [file 12864_2016_2455_MOESM1_ESM.zip › Supplement/Supplement Figure 1.png]

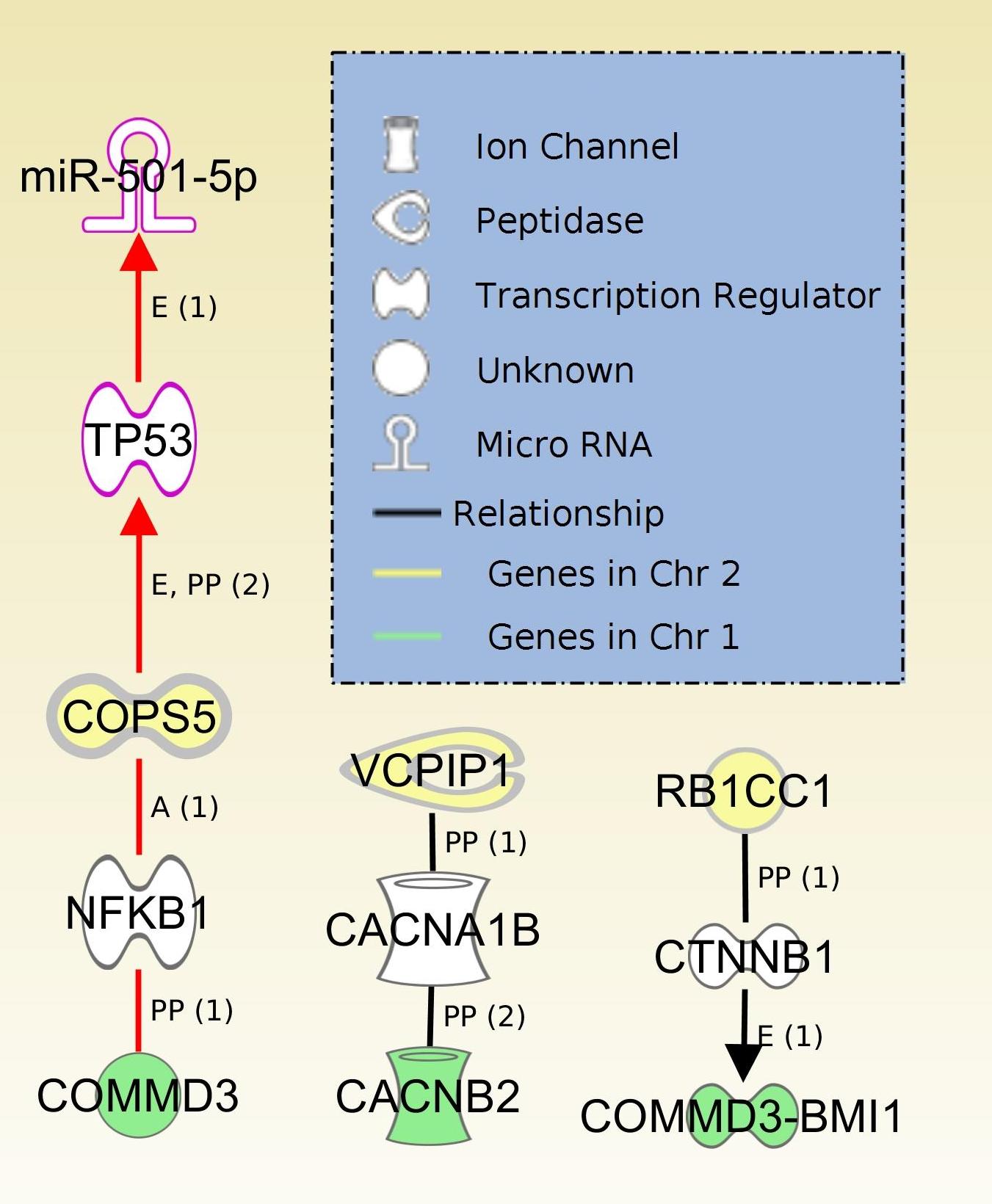

Supplement: Additional file 1: Figure S1. — Flowchart describing the workflow of analysis. The flowchart provides an overview of the analysis performed for understanding regulation of miRNAs and their contribution to the disease phenotype. Figure S2 Interaction network accessed via IPA software for epistasis of miR-501. The graph depicts the interacting genes identified from epistasis scan of miRNA miR-501 in chromosome 1 and chromosome 2. The graph shows all known gene interactions between the two loci where genes colored in yellow are from locus on chromosome 2 and green are from locus on chromosome 1. The red line shows the possible pathway for the regulation of miR-501. Figure S3 qRT-PCR validation of miR-223 expression in EBA and normal murine skin. (ZIP 637 kb) [file 12864_2016_2455_MOESM1_ESM.zip › Supplement/Supplement Figure 2.jpg]
