# Supplementary material for: Dissecting genetics of cutaneous miRNA in a mouse model of an autoimmune blistering disease
Source: BMC Genomics. 2016 Feb 16;17:112. doi: 10.1186/s12864-016-2455-2 (PMC4755013; doi:10.1186/s12864-016-2455-2)
Supplement: Additional file 1: Figure S1. — Flowchart describing the workflow of analysis. The flowchart provides an overview of the analysis performed for understanding regulation of miRNAs and their contribution to the disease phenotype. Figure S2 Interaction network accessed via IPA software for epistasis of miR-501. The graph depicts the interacting genes identified from epistasis scan of miRNA miR-501 in chromosome 1 and chromosome 2. The graph shows all known gene interactions between the two loci where genes colored in yellow are from locus on chromosome 2 and green are from locus on chromosome 1. The red line shows the possible pathway for the regulation of miR-501. Figure S3 qRT-PCR validation of miR-223 expression in EBA and normal murine skin. (ZIP 637 kb) [file 12864_2016_2455_MOESM1_ESM.zip › Supplement/Supplementary 4.doc]

**Supplementary Figure 1:** **Mean haplotype probabilities for individual SNPs across mice in 4th generation.** The figure shows distribution of all 1200 markers (SNPs) across 19 autosomes and X chromosomes for the four progenitor strains (BxD2, NZM2410/J, MRL/MpJ, and CAST/EiJ). The results show that all the SNPs have almost equal distribution in the G4 generation and no bias towards any progenitor strain can be observed.

Supplementary Table 5: List of 1200 SNPs used in eQTL mapping of miRNA

| **SNP** | **Chromosome** | **Position (Mb) NCBI 36** |
| --- | --- | --- |
| rs3683945 | 1 | 1.59369 |
| rs6269442 | 1 | 1.74109 |
| rs13475701 | 1 | 2.258035 |
| rs13475706 | 1 | 2.948225 |
| rs3716083 | 1 | 4.506905 |
| rs3722996 | 1 | 5.62965 |
| rs13475729 | 1 | 6.43605 |
| rs3671256 | 1 | 8.1343 |
| rs13475750 | 1 | 9.73185 |
| rs6404446 | 1 | 10.3871 |
| rs6173215 | 1 | 11.37385 |
| mCV24784983 | 1 | 12.642 |
| rs6166266 | 1 | 12.9743 |
| rs3677683 | 1 | 13.66055 |
| rs3695988 | 1 | 14.887 |
| rs4137502 | 1 | 15.44375 |
| rs3707642 | 1 | 16.2841 |
| rs3681732 | 1 | 17.0939 |
| rs3683997 | 1 | 17.96785 |
| rs4222295 | 1 | 19.5265 |
| gnf01.036.770 | 1 | 20.01525 |
| rs13475827 | 1 | 20.4954 |
| rs13475834 | 1 | 21.26045 |
| CEL.1_44668113 | 1 | 22.19085 |
| rs13475847 | 1 | 22.98455 |
| rs13475851 | 1 | 23.769 |
| CEL.1_49993068 | 1 | 24.8401 |
| rs3724092 | 1 | 25.1484 |
| rs13475866 | 1 | 25.7277 |
| rs6183203 | 1 | 26.7665 |
| rs6386920 | 1 | 27.07625 |
| rs3723035 | 1 | 27.806 |
| rs13475881 | 1 | 29.21965 |
| rs6353774 | 1 | 29.88995 |
| rs3716105 | 1 | 30.7987 |
| rs6322485 | 1 | 31.6518 |
| rs6395308 | 1 | 32.4598 |
| rs6288543 | 1 | 34.27845 |
| rs6191076 | 1 | 36.00035 |
| rs13475919 | 1 | 36.51025 |
| rs3713616 | 1 | 37.244 |
| rs13475931 | 1 | 38.18575 |
| gnf01.075.385 | 1 | 38.95055 |
| rs13475939 | 1 | 39.3421 |
| mCV23431007 | 1 | 40.16115 |
| rs13475946 | 1 | 40.3596 |
| rs3723062 | 1 | 43.9392 |
| UT_1_89.100476 | 1 | 44.54765 |
| rs6195073 | 1 | 45.23955 |
| rs13475980 | 1 | 46.77495 |
| rs6268443 | 1 | 47.63955 |
| rs6157345 | 1 | 48.75805 |
| rs3726669 | 1 | 49.447 |
| rs13476003 | 1 | 49.8411 |
| rs13476012 | 1 | 50.9005 |
| rs6368370 | 1 | 51.873 |
| CEL.1_103251925 | 1 | 52.6235 |
| CEL.1_105423103 | 1 | 53.699 |
| rs13476036 | 1 | 54.3775 |
| rs3685919 | 1 | 55.764 |
| rs13476050 | 1 | 56.254 |
| rs3726043 | 1 | 56.827 |
| rs13476061 | 1 | 57.879 |
| mCV24201027 | 1 | 58.8135 |
| rs3674655 | 1 | 59.6235 |
| rs3695581 | 1 | 60.3185 |
| rs3678634 | 1 | 61.252 |
| rs3717360 | 1 | 62.4485 |
| rs3694822 | 1 | 62.871 |
| rs6228473 | 1 | 64.262 |
| rs3699561 | 1 | 66.4945 |
| rs3724826 | 1 | 66.701 |
| rs13476119 | 1 | 68.0535 |
| rs8250053 | 1 | 68.526 |
| rs6279930 | 1 | 69.6375 |
| gnf01.137.841 | 1 | 70.048 |
| rs13476141 | 1 | 71.215 |
| rs6382880 | 1 | 72.209 |
| rs13476152 | 1 | 72.98 |
| rs13476163 | 1 | 74.359 |
| rs6411476 | 1 | 75.4135 |
| UT_1_113.684537 | 1 | 78.95 |
| rs6387609 | 1 | 79.915 |
| rs13476208 | 1 | 81.978 |
| rs13476229 | 1 | 85.6175 |
| rs8245216 | 1 | 86.5855 |
| rs13466711 | 1 | 87.0325 |
| rs3707910 | 1 | 87.2105 |
| rs3700831 | 1 | 88.973 |
| rs13476265 | 1 | 90.7705 |
| rs13474399 | 1 | 91.2655 |
| rs13476273 | 1 | 92.0105 |
| rs6157620 | 1 | 92.693 |
| mCV22849619 | 1 | 94.3545 |
| rs3667164 | 1 | 95.2555 |
| rs3713997 | 2 | 1.575535 |
| rs4136817 | 2 | 2.11558 |
| gnf02.006.767 | 2 | 4.875595 |
| rs6240512 | 2 | 5.45 |
| rs6287980 | 2 | 6.8199 |
| CZECH.2_15618849 | 2 | 7.7947 |
| rs6402127 | 2 | 8.86095 |
| rs13476366 | 2 | 9.66025 |
| rs3674936 | 2 | 10.14665 |
| rs6286688 | 2 | 12.1953 |
| rs13476391 | 2 | 12.97545 |
| rs13476399 | 2 | 14.0723 |
| gnf02.035.469 | 2 | 16.925 |
| rs13476439 | 2 | 18.98325 |
| rs13476454 | 2 | 20.5698 |
| rs13476467 | 2 | 22.1286 |
| rs13476472 | 2 | 22.7743 |
| rs6265423 | 2 | 23.51 |
| rs6250599 | 2 | 24.26445 |
| CEL.2_50605053 | 2 | 25.25025 |
| rs3725341 | 2 | 25.66055 |
| rs13476503 | 2 | 26.52125 |
| rs3718711 | 2 | 28.19405 |
| rs13476530 | 2 | 29.9281 |
| rs4223189 | 2 | 30.819 |
| CEL.2_63143553 | 2 | 31.52145 |
| rs6222797 | 2 | 32.4785 |
| rs13476553 | 2 | 33.40295 |
| rs6381994 | 2 | 34.04385 |
| rs3664661 | 2 | 35.71825 |
| CEL.2_73370728 | 2 | 36.65725 |
| rs13476580 | 2 | 37.3599 |
| gnf02.076.311 | 2 | 38.7465 |
| CEL.2_79237503 | 2 | 39.59175 |
| rs3722345 | 2 | 40.22785 |
| rs3667007 | 2 | 40.9731 |
| rs8273639 | 2 | 42.3062 |
| rs3689658 | 2 | 42.7594 |
| rs13476621 | 2 | 43.59655 |
| rs6404809 | 2 | 44.4227 |
| rs13476636 | 2 | 45.74265 |
| rs13476639 | 2 | 46.33345 |
| rs13476649 | 2 | 47.93795 |
| rs6316774 | 2 | 48.36695 |
| rs6406705 | 2 | 50.1 |
| gnf02.102.146 | 2 | 51.87 |
| rs13476689 | 2 | 53.6525 |
| rs13476691 | 2 | 54.1725 |
| CEL.2_111981058 | 2 | 55.965 |
| rs4138562 | 2 | 57.103 |
| rs3699172 | 2 | 57.8575 |
| rs8279354 | 2 | 59.496 |
| rs3658927 | 2 | 60.3855 |
| rs3723643 | 2 | 61.072 |
| rs6228179 | 2 | 61.6465 |
| rs6401493 | 2 | 62.5615 |
| gnf02.126.027 | 2 | 63.813 |
| rs3661596 | 2 | 64.985 |
| rs4223511 | 2 | 65.9095 |
| rs13476783 | 2 | 66.8435 |
| CEL.2_135876979 | 2 | 67.977 |
| rs8246404 | 2 | 68.352 |
| rs13476810 | 2 | 71.113 |
| rs6363071 | 2 | 72.929 |
| rs6209325 | 2 | 73.9405 |
| rs6246342 | 2 | 74.844 |
| gnf02.149.271 | 2 | 75.4315 |
| rs6247960 | 2 | 76.202 |
| rs13476860 | 2 | 78.0465 |
| rs13476874 | 2 | 79.7475 |
| rs3664408 | 2 | 80.603 |
| rs3671849 | 2 | 81.489 |
| rs13476889 | 2 | 82.0665 |
| rs3726974 | 2 | 84.8335 |
| rs13476917 | 2 | 85.607 |
| rs13476928 | 2 | 86.96 |
| rs13476932 | 2 | 87.341 |
| rs13476934 | 2 | 89.181 |
| rs6187766 | 2 | 89.6285 |
| rs3679483 | 2 | 89.9305 |
| rs13476950 | 3 | 1.89266 |
| rs13476953 | 3 | 2.181205 |
| rs13476957 | 3 | 2.81451 |
| rs13476963 | 3 | 3.83986 |
| rs13476969 | 3 | 4.845655 |
| rs13476973 | 3 | 5.5079 |
| rs6248752 | 3 | 6.05045 |
| rs13476985 | 3 | 7.4439 |
| rs3659988 | 3 | 8.1751 |
| rs6235984 | 3 | 10.71515 |
| rs3715834 | 3 | 13.2692 |
| rs13477030 | 3 | 13.75005 |
| rs3660588 | 3 | 15.1138 |
| rs13477043 | 3 | 15.6897 |
| rs6246699 | 3 | 18.10395 |
| rs6324747 | 3 | 19.96565 |
| rs3661720 | 3 | 20.9053 |
| rs13477083 | 3 | 21.6649 |
| CEL.3_45598106 | 3 | 22.70045 |
| CEL.3_46558020 | 3 | 23.17895 |
| rs4139913 | 3 | 25.20345 |
| rs6241331 | 3 | 26.19715 |
| rs6335414 | 3 | 26.41085 |
| rs4223969 | 3 | 27.4266 |
| rs13477126 | 3 | 28.22305 |
| rs6301139 | 3 | 29.14685 |
| rs13477138 | 3 | 29.84025 |
| rs13477143 | 3 | 30.3723 |
| rs13477154 | 3 | 31.7219 |
| gnf03.063.824 | 3 | 33.24435 |
| rs13477178 | 3 | 34.77435 |
| rs6198234 | 3 | 34.90985 |
| rs6264454 | 3 | 35.66035 |
| rs13477201 | 3 | 37.62775 |
| rs13477210 | 3 | 38.52775 |
| rs13477217 | 3 | 39.25185 |
| rs13477218 | 3 | 39.8659 |
| rs13477233 | 3 | 41.6416 |
| rs3719390 | 3 | 42.61115 |
| rs6376008 | 3 | 43.23255 |
| rs13477251 | 3 | 44.32925 |
| rs6211610 | 3 | 45.01285 |
| rs13477261 | 3 | 45.9614 |
| rs13477268 | 3 | 46.5693 |
| rs13477272 | 3 | 47.74395 |
| rs3022964 | 3 | 48.5416 |
| rs3726226 | 3 | 49.41765 |
| rs3162061 | 3 | 50.262 |
| rs4138887 | 3 | 51.2465 |
| rs3701653 | 3 | 51.719 |
| rs13477309 | 3 | 52.6365 |
| rs13477321 | 3 | 54.4975 |
| rs3710419 | 3 | 57.678 |
| rs3708412 | 3 | 58.6785 |
| rs3659836 | 3 | 60.6615 |
| rs13477379 | 3 | 61.2705 |
| rs13477385 | 3 | 62.407 |
| rs3671119 | 3 | 63.058 |
| rs3670168 | 3 | 63.9975 |
| rs13477408 | 3 | 65.651 |
| CEL.3_133070717 | 3 | 66.0945 |
| rs13477421 | 3 | 66.9525 |
| rs3676039 | 3 | 67.94 |
| rs13477439 | 3 | 68.8645 |
| rs6407142 | 3 | 71.3605 |
| rs3710548 | 3 | 72.966 |
| rs3657112 | 3 | 74.014 |
| rs13477487 | 3 | 74.909 |
| rs13477494 | 3 | 75.6135 |
| rs13477506 | 3 | 77.2515 |
| gnf03.160.599 | 3 | 78.4015 |
| CEL.3_159340478 | 3 | 79.187 |
| rs3695715 | 4 | 1.82486 |
| gnf04.002.008 | 4 | 2.50674 |
| gnf04.002.599 | 4 | 2.80315 |
| rs3660863 | 4 | 3.563665 |
| UT_4_10.84692 | 4 | 5.41305 |
| rs13477568 | 4 | 6.88245 |
| mCV22939387 | 4 | 7.6607 |
| rs6287606 | 4 | 9.02265 |
| CEL.4_19508304 | 4 | 9.75415 |
| rs13477596 | 4 | 10.33335 |
| mCV22919271 | 4 | 12.0754 |
| rs13477617 | 4 | 13.4431 |
| rs3701432 | 4 | 14.59515 |
| rs3663744 | 4 | 15.4556 |
| rs3674908 | 4 | 16.1483 |
| rs13477643 | 4 | 17.46495 |
| rs3684104 | 4 | 19.13495 |
| rs13477662 | 4 | 19.8689 |
| CEL.4_40541402 | 4 | 20.19845 |
| rs3698283 | 4 | 21.5988 |
| rs13477676 | 4 | 22.4397 |
| rs13477681 | 4 | 23.09405 |
| rs3663355 | 4 | 24.06305 |
| rs3676423 | 4 | 25.90155 |
| rs3677770 | 4 | 27.36615 |
| rs6239799 | 4 | 28.255 |
| rs13477726 | 4 | 28.88765 |
| rs3712541 | 4 | 29.92405 |
| rs3690581 | 4 | 31.1841 |
| rs6254381 | 4 | 31.67325 |
| rs13477749 | 4 | 33.0723 |
| rs13477765 | 4 | 35.44335 |
| rs6222684 | 4 | 35.9006 |
| rs13477774 | 4 | 36.601 |
| rs13477785 | 4 | 37.8874 |
| rs3708471 | 4 | 38.25825 |
| CEL.4_77952063 | 4 | 39.6618 |
| rs4139273 | 4 | 40.67045 |
| rs3676928 | 4 | 41.2055 |
| rs3699276 | 4 | 41.41715 |
| rs6209043 | 4 | 44.8031 |
| rs6271003 | 4 | 46.3225 |
| rs3659791 | 4 | 47.80725 |
| rs13477860 | 4 | 48.5054 |
| rs13477873 | 4 | 50.5515 |
| rs6315002 | 4 | 51.361 |
| rs13477883 | 4 | 51.8175 |
| rs6324470 | 4 | 52.8255 |
| rs6226080 | 4 | 54.1915 |
| rs3709486 | 4 | 55.6475 |
| rs6381371 | 4 | 57.8545 |
| rs3692563 | 4 | 59.0325 |
| rs3675629 | 4 | 60.3655 |
| rs13477952 | 4 | 61.3145 |
| rs13477959 | 4 | 61.9365 |
| gnf04.123.367 | 4 | 63.8535 |
| rs6355837 | 4 | 64.6955 |
| UT_4_132.137715 | 4 | 66.404 |
| rs3663950 | 4 | 67.6425 |
| rs13478002 | 4 | 68.115 |
| rs3688566 | 4 | 70.563 |
| rs3719891 | 4 | 71.0475 |
| rs3023025 | 4 | 71.386 |
| rs6230717 | 4 | 73.637 |
| rs6268364 | 4 | 75.695 |
| rs13478089 | 4 | 76.902 |
| rs13478068 | 4 | 77.296 |
| rs6279100 | 4 | 77.779 |
| rs13478094 | 5 | 2.152025 |
| rs3664617 | 5 | 2.23405 |
| rs6402980 | 5 | 2.71599 |
| rs6307393 | 5 | 3.82206 |
| rs13478110 | 5 | 4.870565 |
| rs3714258 | 5 | 6.18555 |
| rs6353097 | 5 | 7.4025 |
| rs3692039 | 5 | 8.68325 |
| rs13478133 | 5 | 10.56 |
| rs13478139 | 5 | 11.2599 |
| rs6349956 | 5 | 12.1887 |
| CEL.5_24211033 | 5 | 12.6518 |
| rs13478154 | 5 | 13.7204 |
| rs3696671 | 5 | 14.018 |
| rs3680434 | 5 | 14.54475 |
| rs13469943 | 5 | 15.6901 |
| rs3700706 | 5 | 16.48335 |
| rs3673363 | 5 | 17.93075 |
| rs6341620 | 5 | 18.74635 |
| rs6256504 | 5 | 20.0778 |
| rs13478205 | 5 | 20.71695 |
| rs6215373 | 5 | 22.3474 |
| rs3659933 | 5 | 23.2353 |
| CEL.5_45872918 | 5 | 23.81945 |
| rs3714001 | 5 | 25.5436 |
| rs3664008 | 5 | 27.0241 |
| mCV23125912 | 5 | 27.8076 |
| CEL.5_56167948 | 5 | 28.96195 |
| rs3090667 | 5 | 30.3118 |
| rs6354160 | 5 | 30.9488 |
| mCV23386455 | 5 | 32.2904 |
| rs4225248 | 5 | 33.52665 |
| rs13478309 | 5 | 33.76915 |
| gnf05.061.650 | 5 | 33.8696 |
| rs3695107 | 5 | 35.40285 |
| rs6340166 | 5 | 36.5941 |
| rs6161105 | 5 | 37.3956 |
| rs13478337 | 5 | 38.93725 |
| rs13478352 | 5 | 40.7797 |
| rs3721607 | 5 | 41.46715 |
| rs3667334 | 5 | 41.7355 |
| rs13459087 | 5 | 43.7605 |
| rs3673049 | 5 | 45.0583 |
| rs13478402 | 5 | 48.3098 |
| CEL.5_93945748 | 5 | 48.53385 |
| rs3661241 | 5 | 49.13315 |
| rs3705458 | 5 | 49.86485 |
| rs13478433 | 5 | 52.179 |
| rs13478451 | 5 | 54.542 |
| rs13459186 | 5 | 55.267 |
| UT_5_110.343536 | 5 | 55.889 |
| gnf05.106.197 | 5 | 56.2515 |
| rs13478473 | 5 | 57.3305 |
| rs13478483 | 5 | 59.203 |
| CEL.5_117374791 | 5 | 60.169 |
| rs8239888 | 5 | 61.009 |
| rs13478496 | 5 | 61.342 |
| rs13478501 | 5 | 62.1205 |
| rs3671202 | 5 | 62.645 |
| gnf05.120.129 | 5 | 63.297 |
| rs13478518 | 5 | 64.1325 |
| rs13478527 | 5 | 64.8825 |
| gnf05.124.386 | 5 | 65.4245 |
| rs13478540 | 5 | 67.2605 |
| rs13478546 | 5 | 68.5045 |
| rs4225536 | 5 | 69.449 |
| rs6298689 | 5 | 70.1205 |
| rs3656197 | 5 | 71.129 |
| rs13478574 | 5 | 72.434 |
| rs3718776 | 5 | 75.1965 |
| rs3661828 | 6 | 1.708385 |
| rs13478602 | 6 | 1.898545 |
| rs6172481 | 6 | 2.303965 |
| rs13478617 | 6 | 3.98207 |
| rs13478621 | 6 | 4.565815 |
| rs13478626 | 6 | 5.05525 |
| rs13478631 | 6 | 5.6131 |
| rs13478641 | 6 | 8.00545 |
| rs3655269 | 6 | 8.96125 |
| rs13478649 | 6 | 9.2591 |
| rs13478656 | 6 | 10.9469 |
| rs3684494 | 6 | 12.1828 |
| rs3671709 | 6 | 12.88205 |
| rs13478677 | 6 | 13.95055 |
| gnf06.026.418 | 6 | 14.68045 |
| rs13478697 | 6 | 16.40025 |
| rs13478705 | 6 | 17.2287 |
| rs6330932 | 6 | 18.74085 |
| rs13478719 | 6 | 19.2476 |
| gnf06.037.785 | 6 | 20.3746 |
| rs13478727 | 6 | 21.88835 |
| rs6238771 | 6 | 22.5983 |
| rs13478732 | 6 | 22.69165 |
| rs13478745 | 6 | 24.49635 |
| rs4139698 | 6 | 24.9097 |
| rs13478753 | 6 | 25.5736 |
| rs13478761 | 6 | 26.8508 |
| rs6355719 | 6 | 28.15335 |
| CEL.6_57082524 | 6 | 28.52995 |
| rs13478780 | 6 | 29.7366 |
| rs13478783 | 6 | 30.27065 |
| rs6378343 | 6 | 31.8865 |
| rs13478801 | 6 | 32.52885 |
| rs6195266 | 6 | 34.84475 |
| mhcCD8b4 | 6 | 35.63905 |
| rs13478819 | 6 | 36.62905 |
| rs3699367 | 6 | 38.19475 |
| rs13478841 | 6 | 39.09445 |
| rs6377140 | 6 | 39.8798 |
| rs6181382 | 6 | 40.6995 |
| CEL.6_83434907 | 6 | 41.57365 |
| rs13459097 | 6 | 42.4201 |
| CEL.6_86289708 | 6 | 42.95545 |
| rs13478882 | 6 | 44.80745 |
| rs6239023 | 6 | 47.00295 |
| rs6349084 | 6 | 48.34875 |
| rs13478919 | 6 | 48.93895 |
| rs4138572 | 6 | 49.1817 |
| rs13478943 | 6 | 51.85 |
| rs6208251 | 6 | 52.4195 |
| rs13478952 | 6 | 53.1275 |
| rs3670475 | 6 | 54.951 |
| rs13478971 | 6 | 55.625 |
| rs13478977 | 6 | 56.237 |
| rs6393943 | 6 | 57.1785 |
| rs6401637 | 6 | 58.02 |
| mCV23042866 | 6 | 58.3535 |
| rs13478997 | 6 | 59.011 |
| rs3695724 | 6 | 59.9785 |
| rs13479006 | 6 | 60.6245 |
| gnf06.122.747 | 6 | 62.003 |
| rs13479014 | 6 | 62.4995 |
| rs6389420 | 6 | 63.497 |
| rs3681620 | 6 | 63.977 |
| CEL.6_130920075 | 6 | 65.094 |
| rs3722480 | 6 | 65.629 |
| rs6339546 | 6 | 66.959 |
| rs13479053 | 6 | 67.1005 |
| rs3023102 | 6 | 67.6665 |
| rs13479063 | 6 | 68.17 |
| rs13479071 | 6 | 69.1015 |
| rs3672808 | 6 | 69.903 |
| rs6329892 | 6 | 71.1845 |
| rs3658783 | 6 | 72.132 |
| rs6265387 | 6 | 73.6015 |
| rs3711088 | 6 | 74.13 |
| gnf06.149.306 | 6 | 74.607 |
| rs3700068 | 7 | 2.093775 |
| rs3714915 | 7 | 2.159215 |
| rs6384973 | 7 | 2.518405 |
| rs3659551 | 7 | 3.45475 |
| mCV23738426 | 7 | 5.3746 |
| CEL.7_5627457 | 7 | 6.14855 |
| rs3658362 | 7 | 6.8311 |
| rs13479139 | 7 | 8.5736 |
| rs6361142 | 7 | 9.1173 |
| rs3689218 | 7 | 9.80455 |
| gnf07.013.809 | 7 | 9.99415 |
| CEL.7_17114061 | 7 | 14.4701 |
| rs4226499 | 7 | 14.80855 |
| rs3147860 | 7 | 15.6518 |
| rs3719311 | 7 | 17.509 |
| rs13479191 | 7 | 18.45855 |
| gnf07.032.889 | 7 | 24.0902 |
| rs4232449 | 7 | 24.29085 |
| rs8255275 | 7 | 26.71115 |
| rs8260975 | 7 | 26.84135 |
| CEL.7_36725559 | 7 | 28.2689 |
| rs3699938 | 7 | 29.13695 |
| rs13479251 | 7 | 29.8301 |
| rs3718641 | 7 | 30.4811 |
| rs3663313 | 7 | 31.694 |
| mCV23672419 | 7 | 32.4148 |
| rs13479277 | 7 | 32.9823 |
| mCV23423763 | 7 | 34.0559 |
| rs3679779 | 7 | 35.77255 |
| rs3705155 | 7 | 37.83375 |
| rs13479319 | 7 | 38.47065 |
| rs13479324 | 7 | 38.99435 |
| rs3676254 | 7 | 39.91555 |
| rs13479338 | 7 | 40.6019 |
| rs13479347 | 7 | 41.71625 |
| rs13479355 | 7 | 42.71555 |
| rs13479358 | 7 | 43.4215 |
| rs13479363 | 7 | 44.1415 |
| rs6213614 | 7 | 44.71525 |
| rs13479375 | 7 | 45.7087 |
| rs13479385 | 7 | 47.1413 |
| rs13479395 | 7 | 48.6044 |
| CEL.7_77850273 | 7 | 48.88295 |
| rs4226783 | 7 | 50.0405 |
| rs13479414 | 7 | 51.3255 |
| rs3699086 | 7 | 51.6255 |
| rs3707067 | 7 | 52.9345 |
| rs13479427 | 7 | 53.5885 |
| rs3713052 | 7 | 54.459 |
| rs6357312 | 7 | 54.695 |
| rs3673653 | 7 | 57.11 |
| rs6386601 | 7 | 57.373 |
| rs13479455 | 7 | 58.301 |
| rs13479461 | 7 | 59.546 |
| rs6194926 | 7 | 60.7545 |
| rs13479477 | 7 | 62.191 |
| rs3023159 | 7 | 63.2135 |
| rs13479506 | 7 | 65.9115 |
| rs13479517 | 7 | 67.567 |
| CEL.7_115892950 | 7 | 67.779 |
| rs13479522 | 7 | 68.0895 |
| rs3716088 | 7 | 70.095 |
| CEL.7_122752866 | 7 | 71.16 |
| rs3663988 | 7 | 73.2525 |
| rs6360601 | 8 | 3.404125 |
| rs6153168 | 8 | 4.149935 |
| rs13479608 | 8 | 5.83375 |
| rs6410533 | 8 | 7.1507 |
| rs13479619 | 8 | 7.63965 |
| rs3657963 | 8 | 8.26825 |
| mCV24845756 | 8 | 11 |
| rs4140004 | 8 | 11.82155 |
| rs3661760 | 8 | 12.27885 |
| rs13479656 | 8 | 13.3214 |
| CEL.8_25963079 | 8 | 14.14285 |
| rs13479673 | 8 | 15.17235 |
| rs4227096 | 8 | 16.18595 |
| rs3706948 | 8 | 16.48775 |
| CEL.8_33812776 | 8 | 17.95815 |
| rs3665028 | 8 | 18.62865 |
| rs3667738 | 8 | 19.7775 |
| rs13479716 | 8 | 20.39725 |
| rs3703811 | 8 | 20.8707 |
| rs3666140 | 8 | 22.0248 |
| rs6386110 | 8 | 22.94865 |
| UT_8_46.498556 | 8 | 23.86235 |
| rs3719401 | 8 | 25.3638 |
| rs13479755 | 8 | 26.01975 |
| CEL.8_51607005 | 8 | 26.76025 |
| rs13479768 | 8 | 27.7208 |
| rs13479776 | 8 | 29.4287 |
| rs3725286 | 8 | 29.9176 |
| rs3707439 | 8 | 30.71445 |
| rs3672639 | 8 | 31.85025 |
| rs13479793 | 8 | 32.2305 |
| rs6372901 | 8 | 33.30195 |
| rs13479807 | 8 | 34.0708 |
| rs13479811 | 8 | 34.78855 |
| rs3656875 | 8 | 35.3603 |
| rs3699406 | 8 | 36.243 |
| rs4227253 | 8 | 38.82545 |
| rs3696502 | 8 | 39.1111 |
| CZECH.8_78291790 | 8 | 40.8463 |
| rs13479863 | 8 | 42.7318 |
| rs13479871 | 8 | 43.79505 |
| rs6257357 | 8 | 44.0357 |
| rs13479880 | 8 | 44.63545 |
| rs13479884 | 8 | 45.09155 |
| rs6391152 | 8 | 46.1889 |
| rs13479922 | 8 | 47.52685 |
| rs4137596 | 8 | 48.6067 |
| rs6285803 | 8 | 49.2506 |
| rs6287320 | 8 | 49.9583 |
| rs13479947 | 8 | 50.915 |
| rs3706149 | 8 | 51.972 |
| rs3669235 | 8 | 52.287 |
| rs13479956 | 8 | 52.428 |
| rs3705275 | 8 | 52.93 |
| gnf08.108.032 | 8 | 54.665 |
| rs3662808 | 8 | 55.968 |
| rs6237645 | 8 | 56.2705 |
| rs13479995 | 8 | 58.1185 |
| rs4227398 | 8 | 58.449 |
| gnf08.118.027 | 8 | 59.666 |
| rs13480014 | 8 | 60.5535 |
| rs3708073 | 8 | 62.142 |
| rs6377872 | 8 | 62.4375 |
| rs6400423 | 8 | 64.553 |
| rs3697596 | 8 | 65.177 |
| mCV23053483 | 9 | 3.42701 |
| rs13480065 | 9 | 4.645205 |
| mCV25073238 | 9 | 5.28645 |
| rs13480071 | 9 | 6.17985 |
| gnf09.012.310 | 9 | 8.9151 |
| rs13480092 | 9 | 9.46335 |
| rs8270115 | 9 | 10.88245 |
| rs3088801 | 9 | 12.4012 |
| rs6404775 | 9 | 13.93645 |
| rs6385855 | 9 | 14.7239 |
| rs3711756 | 9 | 17.13315 |
| UT_9_35.918713 | 9 | 17.50995 |
| rs13480141 | 9 | 18.37725 |
| rs6413270 | 9 | 18.8745 |
| rs13480150 | 9 | 19.7659 |
| rs3714664 | 9 | 20.2575 |
| rs4135590 | 9 | 21.39805 |
| rs13462199 | 9 | 22.2224 |
| rs13480173 | 9 | 23.1683 |
| gnf09.042.496 | 9 | 24.00865 |
| rs13480186 | 9 | 24.99455 |
| rs3723670 | 9 | 26.1614 |
| rs13480208 | 9 | 27.52 |
| rs3666398 | 9 | 28.21735 |
| rs13480217 | 9 | 28.8333 |
| rs3714012 | 9 | 30.5021 |
| gnf09.057.223 | 9 | 31.42595 |
| rs3655717 | 9 | 32.65645 |
| rs6174757 | 9 | 33.9931 |
| rs6355445 | 9 | 34.90505 |
| rs13480271 | 9 | 36.3383 |
| rs6257131 | 9 | 39.2553 |
| gnf09.074.193 | 9 | 39.95145 |
| rs6223687 | 9 | 40.83695 |
| rs3676124 | 9 | 41.5069 |
| rs3695050 | 9 | 42.52465 |
| rs3700596 | 9 | 43.0993 |
| rs3669564 | 9 | 43.89015 |
| gnf09.087.298 | 9 | 45.28895 |
| rs13480345 | 9 | 46.0611 |
| rs13480351 | 9 | 47.08955 |
| CEL.9_95875215 | 9 | 47.96315 |
| rs3689336 | 9 | 48.1564 |
| rs6385971 | 9 | 49.8831 |
| rs3717654 | 9 | 50.9435 |
| rs13480386 | 9 | 51.764 |
| rs3711089 | 9 | 52.697 |
| rs13480399 | 9 | 53.0935 |
| rs13480413 | 9 | 54.8895 |
| rs13480421 | 9 | 55.8805 |
| gnf09.110.002 | 9 | 56.6 |
| rs6320810 | 9 | 57.5325 |
| rs3669563 | 9 | 58.914 |
| rs3721803 | 10 | 1.671245 |
| rs3664101 | 10 | 4.664485 |
| rs4228112 | 10 | 6.36445 |
| rs3686911 | 10 | 7.06155 |
| rs3699409 | 10 | 7.76015 |
| rs3712394 | 10 | 8.83165 |
| rs13480527 | 10 | 9.668 |
| rs3679120 | 10 | 11.3205 |
| rs13480547 | 10 | 12.10365 |
| rs13480554 | 10 | 12.8353 |
| rs13480563 | 10 | 13.92745 |
| gnf10.026.889 | 10 | 14.3387 |
| rs13480570 | 10 | 15.3629 |
| rs13480581 | 10 | 19.34265 |
| rs3716113 | 10 | 21.21085 |
| CEL.10_46671493 | 10 | 23.21025 |
| rs3676667 | 10 | 23.33615 |
| rs6382436 | 10 | 24.2027 |
| rs3696307 | 10 | 26.73145 |
| rs6190748 | 10 | 27.7541 |
| rs6374078 | 10 | 30.284 |
| rs6359581 | 10 | 32.0088 |
| rs3723140 | 10 | 32.9534 |
| rs13480630 | 10 | 33.64185 |
| rs3679902 | 10 | 42.4025 |
| rs13480684 | 10 | 42.6676 |
| CEL.10_86322095 | 10 | 43.0254 |
| rs3089366 | 10 | 44.57185 |
| rs13480702 | 10 | 45.26145 |
| gnf10.091.965 | 10 | 46.2183 |
| rs6175332 | 10 | 49.1609 |
| rs13480734 | 10 | 50.919 |
| rs13480740 | 10 | 51.758 |
| rs6290359 | 10 | 53.714 |
| rs6243755 | 10 | 54.0875 |
| rs13480773 | 10 | 57.0895 |
| rs13480775 | 10 | 57.6195 |
| rs6256918 | 10 | 58.4945 |
| mCV24217147 | 10 | 58.6245 |
| rs13480803 | 10 | 61.284 |
| mCV22832306 | 10 | 62.868 |
| rs3697243 | 10 | 63.125 |
| rs3676330 | 10 | 63.8235 |
| rs13480836 | 11 | 1.72705 |
| rs13480837 | 11 | 1.893425 |
| rs6190775 | 11 | 3.156055 |
| rs13480854 | 11 | 3.76195 |
| rs13480863 | 11 | 4.59676 |
| rs3023249 | 11 | 5.53555 |
| rs13480875 | 11 | 6.09785 |
| CEL.11_14992925 | 11 | 7.49865 |
| rs6313050 | 11 | 7.82615 |
| gnf11.017.294 | 11 | 9.0061 |
| rs3723987 | 11 | 9.6844 |
| rs3023251 | 11 | 10.4266 |
| rs13480910 | 11 | 11.357 |
| rs3723990 | 11 | 13.2611 |
| rs3700830 | 11 | 15.1356 |
| rs13459123 | 11 | 15.4794 |
| rs3723833 | 11 | 16.4756 |
| rs6359329 | 11 | 19.18275 |
| rs6164170 | 11 | 20.05875 |
| rs3701734 | 11 | 20.57055 |
| rs3654344 | 11 | 22.01075 |
| rs13481009 | 11 | 23.1709 |
| rs13481011 | 11 | 23.37095 |
| rs6199956 | 11 | 25.27935 |
| rs3684076 | 11 | 28.11215 |
| rs3697686 | 11 | 29.1905 |
| rs3711357 | 11 | 30.633 |
| rs13481061 | 11 | 31.403 |
| rs13481071 | 11 | 32.5071 |
| rs13481076 | 11 | 33.26615 |
| rs13481084 | 11 | 34.29195 |
| rs6197743 | 11 | 34.9954 |
| mCV24113391 | 11 | 35.6466 |
| rs13481094 | 11 | 36.3239 |
| rs3701609 | 11 | 36.5385 |
| rs13481109 | 11 | 38.2043 |
| rs6259742 | 11 | 41.21065 |
| rs13481127 | 11 | 41.61295 |
| rs3661657 | 11 | 42.2729 |
| rs3719581 | 11 | 43.38615 |
| rs13481145 | 11 | 44.12455 |
| rs3688955 | 11 | 45.19885 |
| rs13481161 | 11 | 46.16125 |
| rs13481173 | 11 | 48.23305 |
| rs3661058 | 11 | 49.39035 |
| rs3090212 | 11 | 49.956 |
| rs6393948 | 11 | 51.731 |
| rs3698446 | 11 | 52.5495 |
| rs6386362 | 11 | 53.42 |
| rs6370458 | 11 | 54.628 |
| rs13481227 | 11 | 55.155 |
| rs3672597 | 11 | 56.0475 |
| rs3699056 | 11 | 56.9325 |
| rs13481247 | 11 | 57.881 |
| rs3712881 | 11 | 60.4645 |
| rs3713137 | 12 | 2.42093 |
| rs13481281 | 12 | 2.86126 |
| rs3678128 | 12 | 3.71036 |
| rs6292954 | 12 | 4.977455 |
| rs13481297 | 12 | 5.3035 |
| rs13481303 | 12 | 5.961 |
| rs3655057 | 12 | 6.6949 |
| rs13481313 | 12 | 7.438 |
| rs3706330 | 12 | 8.2945 |
| rs13481324 | 12 | 8.7906 |
| rs3717860 | 12 | 12.70685 |
| rs13481363 | 12 | 14.33275 |
| rs3089800 | 12 | 14.84265 |
| UT_12_24.561109 | 12 | 15.3027 |
| rs13481371 | 12 | 15.4417 |
| rs6223000 | 12 | 17.43375 |
| rs6311081 | 12 | 18.86865 |
| rs3658100 | 12 | 19.40825 |
| rs13481406 | 12 | 20.33065 |
| rs13481408 | 12 | 20.59145 |
| CEL.12_38788391 | 12 | 22.40755 |
| CEL.12_40646532 | 12 | 23.29255 |
| gnf12.046.238 | 12 | 24.5142 |
| rs13481445 | 12 | 25.7217 |
| rs13481454 | 12 | 26.8103 |
| rs3670749 | 12 | 27.0301 |
| rs3700857 | 12 | 27.5104 |
| rs13481466 | 12 | 28.5536 |
| rs3706319 | 12 | 29.5268 |
| mCV22351241 | 12 | 30.0208 |
| rs13481303.1 | 12 | 32.15175 |
| rs3677344 | 12 | 32.84895 |
| rs6344105 | 12 | 34.43005 |
| rs13481514 | 12 | 35.35235 |
| rs13481521 | 12 | 36.28085 |
| rs13481527 | 12 | 36.99405 |
| rs3655558 | 12 | 37.91315 |
| rs13481541 | 12 | 38.7396 |
| gnf12.077.067 | 12 | 39.8522 |
| rs3662628 | 12 | 40.1812 |
| rs13481556 | 12 | 40.8817 |
| rs13481561 | 12 | 41.6337 |
| rs13481565 | 12 | 42.55895 |
| rs3716095 | 12 | 44.1932 |
| CEL.12_84750094 | 12 | 45.6986 |
| rs3670410 | 12 | 46.2887 |
| rs13481588 | 12 | 46.60355 |
| rs6207869 | 12 | 47.80045 |
| rs13481599 | 12 | 48.67155 |
| rs13481604 | 12 | 49.6586 |
| rs3716084 | 12 | 50.551 |
| rs13481614 | 12 | 51.193 |
| rs13481618 | 12 | 51.8835 |
| rs8273308 | 12 | 52.816 |
| rs13481632 | 12 | 53.817 |
| rs6390948 | 12 | 54.9585 |
| CEL.12_104545022 | 12 | 55.5175 |
| rs13481655 | 12 | 56.6755 |
| rs3023711 | 12 | 58.709 |
| rs3692361 | 12 | 59.4785 |
| rs6329833 | 12 | 60.215 |
| mCV24244050 | 13 | 1.56634 |
| rs6215262 | 13 | 1.89178 |
| rs6301008 | 13 | 3.85141 |
| rs13481683 | 13 | 4.69281 |
| rs13481689 | 13 | 5.4131 |
| rs13481697 | 13 | 6.8637 |
| rs6348604 | 13 | 7.7514 |
| rs13481706 | 13 | 8.21625 |
| rs13481715 | 13 | 9.61545 |
| rs6314295 | 13 | 10.1065 |
| rs3679784 | 13 | 10.4991 |
| rs3663223 | 13 | 11.5885 |
| rs8267104 | 13 | 11.89865 |
| rs6275055 | 13 | 13.0084 |
| CEL.13_27061395 | 13 | 13.88675 |
| rs13481740 | 13 | 15.38465 |
| rs3710348 | 13 | 15.68255 |
| rs3707097 | 13 | 17.136 |
| rs3725187 | 13 | 17.5773 |
| rs13481764 | 13 | 18.3247 |
| gnf13.035.637 | 13 | 18.8054 |
| gnf13.038.133 | 13 | 20.067 |
| rs13481783 | 13 | 21.4677 |
| rs3712907 | 13 | 21.63315 |
| rs3688207 | 13 | 22.7273 |
| rs6244558 | 13 | 23.95565 |
| rs3693942 | 13 | 27.527 |
| rs3720782 | 13 | 28.1654 |
| gnf13.056.787 | 13 | 29.4129 |
| rs3690198 | 13 | 30.40965 |
| rs4229817 | 13 | 33.4907 |
| rs3718727 | 13 | 34.73395 |
| rs13481880 | 13 | 37.2491 |
| rs13481883 | 13 | 37.81985 |
| gnf13.079.671 | 13 | 40.2722 |
| rs13481908 | 13 | 41.10065 |
| mCV24625340 | 13 | 42.4205 |
| rs3686443 | 13 | 43.3313 |
| gnf13.088.732 | 13 | 44.13865 |
| rs3655061 | 13 | 44.8615 |
| rs6288319 | 13 | 45.6377 |
| rs6316213 | 13 | 46.4394 |
| gnf13.093.328 | 13 | 46.74685 |
| rs13481961 | 13 | 49.02005 |
| rs13481983 | 13 | 52.106 |
| rs13481992 | 13 | 53.237 |
| rs13481996 | 13 | 53.801 |
| rs13482005 | 13 | 54.8055 |
| rs13482011 | 13 | 55.7165 |
| rs13482019 | 13 | 56.7795 |
| gnf13.115.241 | 13 | 57.3515 |
| rs6247696 | 13 | 57.9785 |
| rs13482028 | 13 | 58.52 |
| rs13482035 | 13 | 59.4385 |
| rs4230144 | 14 | 4.46196 |
| rs3150398 | 14 | 5.81335 |
| rs13482084 | 14 | 6.53695 |
| rs6290836 | 14 | 6.6892 |
| rs3696385 | 14 | 9.0977 |
| rs3687889 | 14 | 10.25415 |
| rs3692121 | 14 | 12.31495 |
| rs13482104 | 14 | 13.58175 |
| rs6396829 | 14 | 14.407 |
| rs4230248 | 14 | 15.9983 |
| rs13482124 | 14 | 16.24005 |
| rs6313230 | 14 | 16.53965 |
| rs3695383 | 14 | 17.3046 |
| rs13482135 | 14 | 18.1003 |
| rs13482143 | 14 | 20.1892 |
| rs13482148 | 14 | 20.99995 |
| rs13482170 | 14 | 24.5736 |
| mCV24777660 | 14 | 25.424 |
| rs3666583 | 14 | 26.3801 |
| rs13482191 | 14 | 27.63255 |
| rs13482193 | 14 | 28.1207 |
| rs13482194 | 14 | 28.22295 |
| rs13482216 | 14 | 31.2551 |
| rs13482231 | 14 | 33.79855 |
| rs3672425 | 14 | 34.48205 |
| rs13482239 | 14 | 35.08375 |
| rs13482247 | 14 | 36.14835 |
| rs13459144 | 14 | 38.1587 |
| rs13482265 | 14 | 39.71015 |
| rs3023413 | 14 | 40.08355 |
| CEL.14_73162771 | 14 | 40.89025 |
| rs13482272 | 14 | 41.35455 |
| rs6319148 | 14 | 41.9373 |
| rs13482284 | 14 | 43.3079 |
| rs13482296 | 14 | 44.658 |
| rs6395984 | 14 | 45.73515 |
| rs6291434 | 14 | 46.20525 |
| rs13482327 | 14 | 48.8001 |
| rs3692362 | 14 | 50.0875 |
| rs6191117 | 14 | 50.3035 |
| CEL.14_94845626 | 14 | 51.507 |
| rs13482354 | 14 | 53.1435 |
| rs13482356 | 14 | 53.5865 |
| gnf14.103.048 | 14 | 53.9465 |
| rs3708779 | 14 | 55.584 |
| rs13482375 | 14 | 56.04 |
| rs3726052 | 14 | 56.1715 |
| rs6256423 | 14 | 57.0945 |
| rs6169105 | 14 | 58.8285 |
| rs3665550 | 14 | 59.7125 |
| rs13482398 | 14 | 60.1855 |
| rs13482407 | 14 | 61.226 |
| rs13459176 | 15 | 1.614515 |
| rs13482418 | 15 | 1.809505 |
| CEL.15_4222769 | 15 | 2.174575 |
| rs3665826 | 15 | 2.93561 |
| CEL.15_8331158 | 15 | 4.21209 |
| CEL.15_9687257 | 15 | 4.85883 |
| rs13482431 | 15 | 5.62055 |
| rs3711814 | 15 | 6.57455 |
| CEL.15_14786403 | 15 | 7.4065 |
| CEL.15_15482356 | 15 | 7.75345 |
| rs13482455 | 15 | 8.33045 |
| rs13482469 | 15 | 10.43605 |
| rs13482477 | 15 | 11.22445 |
| rs13482482 | 15 | 12.2617 |
| rs13482485 | 15 | 12.69025 |
| rs13482497 | 15 | 14.1257 |
| rs13482504 | 15 | 15.12595 |
| rs6188239 | 15 | 17.13045 |
| CEL.15_36490596 | 15 | 18.18275 |
| rs3695416 | 15 | 19.20705 |
| rs13482529 | 15 | 19.47785 |
| CEL.15_43206205 | 15 | 21.5098 |
| rs13482549 | 15 | 22.73725 |
| rs13482558 | 15 | 23.49905 |
| rs13482571 | 15 | 24.9149 |
| rs3707900 | 15 | 26.1574 |
| rs13482585 | 15 | 26.9336 |
| rs6400804 | 15 | 28.31895 |
| rs13482595 | 15 | 29.224 |
| rs3683495 | 15 | 30.517 |
| rs13482602 | 15 | 30.6302 |
| rs3677062 | 15 | 31.6465 |
| rs6381737 | 15 | 32.7099 |
| rs13482628 | 15 | 33.8543 |
| rs8259436 | 15 | 37.34015 |
| rs3660192 | 15 | 38.82745 |
| rs3665030 | 15 | 39.61175 |
| rs13482673 | 15 | 41.0248 |
| rs3660367 | 15 | 42.2662 |
| rs13482687 | 15 | 42.888 |
| rs13482704 | 15 | 45.1269 |
| rs13482712 | 15 | 46.03815 |
| rs3719217 | 15 | 47.54095 |
| rs6285067 | 15 | 47.57245 |
| rs13482738 | 15 | 49.81235 |
| rs13482744 | 15 | 50.6905 |
| rs13482751 | 15 | 51.391 |
| rs4152638 | 16 | 2.163255 |
| rs4158907 | 16 | 3.98141 |
| rs4160288 | 16 | 4.78508 |
| rs4162800 | 16 | 6.2364 |
| rs4163196 | 16 | 6.7004 |
| rs4165081 | 16 | 9.94155 |
| rs4165440 | 16 | 12.08785 |
| rs4165467 | 16 | 12.70365 |
| rs4165602 | 16 | 13.71815 |
| rs4167031 | 16 | 14.4262 |
| rs4168640 | 16 | 15.49585 |
| rs4170308 | 16 | 16.2359 |
| rs4171932 | 16 | 17.15415 |
| rs4175608 | 16 | 19.59125 |
| rs4177651 | 16 | 20.34165 |
| rs4179075 | 16 | 21.16725 |
| rs4182243 | 16 | 23.02635 |
| rs4183448 | 16 | 23.59875 |
| rs4185639 | 16 | 25.1493 |
| rs4186744 | 16 | 25.72105 |
| rs3696661 | 16 | 25.83835 |
| rs6163640 | 16 | 26.7206 |
| CZECH.16_55104521 | 16 | 27.54665 |
| rs4189277 | 16 | 28.64625 |
| rs4191367 | 16 | 29.56645 |
| rs4193069 | 16 | 30.48905 |
| rs4197150 | 16 | 33.14835 |
| rs4197715 | 16 | 33.6317 |
| rs4198737 | 16 | 34.5058 |
| rs4200124 | 16 | 35.3475 |
| rs4201998 | 16 | 36.2556 |
| rs3718160 | 16 | 38.4339 |
| rs6317052 | 16 | 39.74645 |
| rs4211364 | 16 | 40.4151 |
| rs4211664 | 16 | 41.3908 |
| rs3680665 | 16 | 42.2768 |
| rs4213268 | 16 | 43.23115 |
| rs4214396 | 16 | 43.74945 |
| petM.01322.2 | 16 | 44.0791 |
| rs4217260 | 16 | 46.4999 |
| rs4219905 | 16 | 46.76915 |
| rs3696981 | 16 | 48.8085 |
| rs3712360 | 16 | 49.2692 |
| rs4136382 | 17 | 1.694405 |
| rs13482843 | 17 | 2.022315 |
| UT_17_3.221024 | 17 | 3.01946 |
| rs6196216 | 17 | 4.581645 |
| rs3662820 | 17 | 6.254 |
| rs3672065 | 17 | 7.16815 |
| rs3726555 | 17 | 8.26975 |
| rs13482899 | 17 | 8.8806 |
| rs13482914 | 17 | 10.49135 |
| rs3696835 | 17 | 11.40895 |
| rs3719497 | 17 | 12.81965 |
| rs6397584 | 17 | 13.79415 |
| rs3693494 | 17 | 14.9585 |
| rs3672987 | 17 | 16.62355 |
| rs3682923 | 17 | 17.9855 |
| rs6298471 | 17 | 18.34215 |
| rs6390174 | 17 | 19.5639 |
| rs3702604 | 17 | 20.54585 |
| CEL.17_39772541 | 17 | 20.8807 |
| rs13482997 | 17 | 21.98075 |
| rs6409750 | 17 | 23.7392 |
| rs3700227 | 17 | 26.3754 |
| rs3714226 | 17 | 27.70465 |
| rs3715723 | 17 | 29.40515 |
| gnf17.064.100 | 17 | 31.55575 |
| rs6402097 | 17 | 32.03085 |
| rs3727008 | 17 | 34.0417 |
| rs3660112 | 17 | 35.4717 |
| rs3675634 | 17 | 35.79535 |
| rs3691628 | 17 | 36.37445 |
| rs13483103 | 17 | 37.30205 |
| rs6229946 | 17 | 37.7952 |
| rs3663088 | 17 | 38.72405 |
| rs13483117 | 17 | 39.6201 |
| gnf17.082.284 | 17 | 40.67575 |
| gnf17.083.842 | 17 | 41.45435 |
| rs13483135 | 17 | 41.93865 |
| rs13483144 | 17 | 43.1426 |
| rs4231720 | 17 | 43.6046 |
| rs13483157 | 17 | 44.64385 |
| rs3706382 | 17 | 47.26515 |
| rs13483183 | 18 | 1.75822 |
| rs13483184 | 18 | 1.89822 |
| gnf18.001.688 | 18 | 2.321495 |
| CEL.18_5565618 | 18 | 2.69757 |
| rs3660245 | 18 | 4.63675 |
| rs4231742 | 18 | 5.5424 |
| rs13483212 | 18 | 6.13455 |
| mCV23617245 | 18 | 7.3445 |
| rs6361186 | 18 | 7.5833 |
| rs6358426 | 18 | 8.28655 |
| rs13483244 | 18 | 10.49135 |
| rs4138020 | 18 | 11.3763 |
| gnf18.022.796 | 18 | 12.7349 |
| rs13483263 | 18 | 13.29995 |
| rs13483271 | 18 | 14.5581 |
| rs3722973 | 18 | 15.5476 |
| rs3718586 | 18 | 16.64585 |
| rs3725637 | 18 | 17.29405 |
| rs13483299 | 18 | 18.1398 |
| rs3664831 | 18 | 18.7269 |
| rs3699816 | 18 | 19.73665 |
| rs13483319 | 18 | 20.5338 |
| rs3676196 | 18 | 21.5417 |
| rs4231834 | 18 | 22.09075 |
| CEL.18_46082735 | 18 | 22.89905 |
| rs6184541 | 18 | 23.79935 |
| rs3674314 | 18 | 25.67695 |
| gnf18.051.412 | 18 | 26.63125 |
| rs13483368 | 18 | 27.30575 |
| rs3683378 | 18 | 27.9224 |
| rs6328845 | 18 | 28.9819 |
| CEL.18_60214752 | 18 | 29.92935 |
| rs3720827 | 18 | 31.7249 |
| rs3688789 | 18 | 32.33695 |
| rs3691542 | 18 | 33.3172 |
| rs3658163 | 18 | 34.41045 |
| rs13483426 | 18 | 35.14165 |
| rs6161154 | 18 | 35.8048 |
| rs13483436 | 18 | 36.79595 |
| rs13459193 | 18 | 37.44785 |
| rs13483446 | 18 | 38.44975 |
| rs3706601 | 18 | 38.8543 |
| rs3720876 | 18 | 40.0677 |
| rs13483462 | 18 | 40.6935 |
| rs13483466 | 18 | 41.20495 |
| mCV24836796 | 18 | 42.8447 |
| rs13483482 | 18 | 43.13615 |
| rs4137441 | 18 | 44.40165 |
| rs3671671 | 19 | 2.2098 |
| rs3713033 | 19 | 2.514845 |
| CEL.19_5283144 | 19 | 2.74707 |
| rs6236348 | 19 | 3.10586 |
| rs13483525 | 19 | 5.2592 |
| UT_19_10.709331 | 19 | 5.39435 |
| rs6316813 | 19 | 5.6983 |
| rs3686467 | 19 | 8.47795 |
| rs6223359 | 19 | 9.29985 |
| gnf19.017.711 | 19 | 10.0396 |
| rs6372656 | 19 | 10.9588 |
| rs6309315 | 19 | 11.8306 |
| rs3720897 | 19 | 12.59815 |
| rs6293693 | 19 | 13.0804 |
| rs3090325 | 19 | 13.403 |
| UT_19_29.979736 | 19 | 15.1568 |
| rs13459194 | 19 | 16.0617 |
| rs6237466 | 19 | 16.2189 |
| CEL.19_34542259 | 19 | 17.6972 |
| gnf19.035.019 | 19 | 18.65895 |
| rs3710829 | 19 | 19.12145 |
| rs3655407 | 19 | 20.167 |
| rs3656289 | 19 | 21.24355 |
| rs3687275 | 19 | 21.5723 |
| rs13483643 | 19 | 22.69305 |
| mCV23390953 | 19 | 23.04505 |
| rs3023496 | 19 | 24.10615 |
| rs6194426 | 19 | 25.1017 |
| mCV23069572 | 19 | 26.4381 |
| rs6304326 | 19 | 26.75625 |
| rs13483682 | 19 | 27.618 |
| rs6211533 | 19 | 28.5334 |
| rs6191324 | 19 | 29.6981 |
| UT_4_59.889271 | 19 | 30.0787 |
| rs13483712 | X | 4.56469 |
| CEL.X_8334947 | X | 4.790125 |
| rs13483724 | X | 16.774 |
| gnfX.023.543 | X | 18.1264 |
| gnfX.026.801 | X | 19.7889 |
| CEL.X_39961894 | X | 22.48335 |
| rs13483738 | X | 22.75765 |
| rs13483746 | X | 23.8885 |
| gnfX.035.350 | X | 24.14275 |
| rs13483750 | X | 24.3639 |
| CEL.X_44311522 | X | 24.65935 |
| rs13483756 | X | 25.15205 |
| rs13483761 | X | 26.7345 |
| rs13483765 | X | 27.3938 |
| gnfX.044.260 | X | 29.03805 |
| rs13483777 | X | 29.2142 |
| rs13483781 | X | 29.81445 |
| rs13483783 | X | 30.44875 |
| rs13483786 | X | 30.93435 |
| petM.05810.1 | X | 33.45785 |
| rs3157124 | X | 34.34705 |
| rs13483825 | X | 35.7264 |
| rs13483831 | X | 36.4868 |
| CEL.X_68179178 | X | 36.9337 |
| rs13483844 | X | 37.97345 |
| CEL.X_72627341 | X | 39.15845 |
| CEL.X_72875547 | X | 39.28255 |
| rs13483858 | X | 39.7231 |
| rs13483862 | X | 40.13485 |
| CEL.X_75125049 | X | 40.4097 |
| rs13483868 | X | 40.9212 |
| rs3725966 | X | 41.3719 |
| CEL.X_77780392 | X | 41.73735 |
| rs13483877 | X | 42.0969 |
| rs13483805 | X | 43.72005 |
| gnfX.076.619 | X | 44.96035 |
| rs13483884 | X | 45.57505 |
| gnfX.080.189 | X | 46.6892 |
| rs13483894 | X | 46.84715 |
| rs6182892 | X | 47.0858 |
| CEL.X_89752158 | X | 47.7719 |
| CEL.X_91222960 | X | 48.5073 |
| gnfX.084.751 | X | 49.01365 |
| gnfX.086.039 | X | 49.66095 |
| rs13483921 | X | 50.4275 |
| rs13483926 | X | 51.642 |
| rs13483929 | X | 52.0085 |
| CEL.X_100299803 | X | 53.0455 |
| rs13483937 | X | 53.9005 |
| rs13483941 | X | 54.3155 |
| rs6342003 | X | 54.8305 |
| CEL.X_106858553 | X | 56.3185 |
| rs6363550 | X | 57.6805 |
| rs13483971 | X | 58.459 |
| CEL.X_112457192 | X | 59.12 |
| CEL.X_112904656 | X | 59.3435 |
| CZECH.X_116527625 | X | 61.4965 |
| rs6205221 | X | 63.415 |
| rs6221690 | X | 63.786 |
| rs13483997 | X | 64.324 |
| rs13484004 | X | 65.155 |
| rs3702256 | X | 65.742 |
| CEL.X_125736335 | X | 66.132 |
| rs3697198 | X | 66.3755 |
| rs13484024 | X | 67.2705 |
| rs13484031 | X | 68.1875 |
| rs13484034 | X | 68.6065 |
| rs13484043 | X | 69.6215 |
| CEL.X_133525088 | X | 70.034 |
| rs13484050 | X | 70.485 |
| rs13484070 | X | 73.5715 |
| CEL.X_143595976 | X | 75.0715 |
| rs13484087 | X | 76.1915 |
| rs13484094 | X | 76.8715 |
| rs6365259 | X | 78.3085 |
| CEL.X_154048891 | X | 80.2585 |
| CEL.X_155542834 | X | 80.934 |
| gnfX.146.867 | X | 81.562 |
